# Patient characteristics associated with the need for high doses of tenapanor hydrochloride in dialysis patients with hyperphosphatemia: A pooled analysis of three clinical trials

**DOI:** 10.1371/journal.pone.0356873

**Published:** 2026-08-28

**Authors:** Nobuo Nagano, Shin Tokunaga, Shinji Asada, Masafumi Fukagawa, Tadao Akizawa

**Affiliations:** 1 Kidney Disease and Dialysis Center, Hidaka Hospital, Hidaka-kai, Gunma, Japan; 2 Department of Medicine, Tokyo Women's Medical University Adachi Medical Center, Tokyo, Japan; 3 Medical Affairs Department, Kyowa Kirin Co., Ltd., Tokyo, Japan; 4 Division of Nephrology, Endocrinology, and Metabolism, Department of Internal Medicine, Tokai University School of Medicine, Kanagawa, Japan; 5 Division of Nephrology, Department of Medicine, Showa Medical University School of Medicine, Tokyo, Japan; Showa University, JAPAN

## Abstract

Tenapanor hydrochloride (tenapanor), a selective inhibitor of the sodium/hydrogen exchanger isoform 3, is approved for management of hyperphosphatemia in patients undergoing dialysis. Dosage is initiated at 5 mg twice daily and can be titrated to 10, 20, or 30 mg twice daily according to clinical response and tolerability. However, patient characteristics associated with the need for higher tenapanor doses remain unclear. This study is a pooled analysis of data from three clinical trials conducted in Japan, in which tenapanor dose was titrated starting from 5 mg twice daily. After 8 weeks of treatment, dialysis patients were retrospectively categorized into a low-dose group (5 or 10 mg/dose; n = 127) and a high-dose group (20 or 30 mg/dose; n = 85) according to their final dose. Univariate and multivariate analyses were performed to identify baseline factors independently associated with higher tenapanor dose requirements. At Week 8, mean ± standard deviation tenapanor dose was 8.0 ± 2.5 mg twice daily in the low-dose group, and 26.7 ± 4.9 mg twice daily in the high-dose group. Serum phosphorus levels decreased gradually over 8 weeks in both groups; however, no significant between-group difference in change from baseline was observed. Baseline comparisons showed significant differences in serum phosphorus, serum tartrate-resistant acid phosphatase 5b (TRACP-5b), number of concomitant phosphate binders, use of sevelamer hydrochloride and laxatives, and Bristol Stool Form Scale (BSFS) scores. Multivariate analysis identified serum TRACP-5b as a significant positive independent predictor and BSFS score as a significant negative independent predictor for requiring higher doses of tenapanor. Elevated TRACP-5b levels and lower BSFS scores were associated with a requirement for higher doses of tenapanor. Incorporating bone turnover and bowel habit assessments into clinical evaluations may support individualized dosing strategies. Large-scale prospective studies in real-world settings are warranted to confirm these findings.

**Trial registration:**

The original clinical trials were registered at ClinicalTrials.gov under the identifiers NCT04767581, NCT04766398, and NCT04766385.

## Introduction

In hemodialysis (HD) patients, the loss of urinary phosphate (P) excretion makes dialysis alone insufficient to eliminate excess P, often resulting in hyperphosphatemia. This condition contributes to the development and progression of cardiovascular calcification and secondary hyperparathyroidism, which are associated with increased risks of all-cause mortality, cardiovascular mortality, and fractures [1–3]. To prevent hyperphosphatemia in HD patients, fundamental strategies include ensuring adequate dialysis clearance, providing dietary and nutritional education, and implementing pharmacological interventions.

For many years, phosphate binders (PBs), which bind dietary P in the gastrointestinal (GI) tract and promote its fecal excretion, have been the only approved pharmacological treatment for hyperphosphatemia. However, PBs are often associated with adverse effects, the use of two or more PBs in combination, and the requirement for frequent daily dosing [3–6]. In addition, they substantially contribute to high pill burden, which in turn leads to decreased medication adherence and poor P control [7–11].

In humans, ingested P is mainly absorbed via the paracellular pathway between epithelial cells in the upper small intestine, and the amount absorbed increases linearly with intraluminal P concentration (i.e., the amount of P ingested). Tenapanor hydrochloride (tenapanor), which was newly launched in the USA in 2023, and in Japan in 2024, is a novel therapeutic agent for hyperphosphatemia in dialysis patients [12]. It selectively inhibits the sodium/hydrogen exchanger isoform 3, which is expressed on the apical membrane of intestinal epithelial cells, and through this action reduces paracellular P absorption [13,14]. Several clinical trials have been conducted in Japanese dialysis patients, and the efficacy of tenapanor in lowering serum phosphorus levels and reducing the number of PB pills has already been demonstrated [15–21].

The dosing regimen approved in Japan recommends initiating treatment at 5 mg twice daily, with titration to 10, 20, or 30 mg twice daily based on clinical response (serum phosphorus levels) and tolerability, with dose increases made at intervals of at least 1 week [12]. However, tenapanor is frequently associated with GI side effects, such as diarrhea, soft stools, and increased stool frequency [12], which may make patients reluctant to escalate the dose in real-world clinical practice. Nevertheless, the patient characteristics associated with the need for and the ability to tolerate higher doses of tenapanor remain unclear.

In the present study, we conducted a pooled analysis of data from three clinical trials [18–20], through which we identified patient background factors that necessitate and enable the use of higher tenapanor doses.

## Materials and methods

The present study is a pooled analysis using data from three phase 3 clinical trials (Trials A, B, and C) carried out in Japan [18–20]. The original clinical trials were conducted in accordance with the Declaration of Helsinki, the Pharmaceuticals and Medical Devices Act, and Good Clinical Practice (Ministry of Health and Welfare Ordinance No. 28, March 27, 1997, as amended). Ethical approval of each clinical trial [18–20] was obtained from the ethics committees of all 94 participating institutions, and written informed consent was obtained from each patient prior to participation. This pooled post hoc analysis was approved by the ethics committee of Kyowa Kirin Co., Ltd. (approval number: MA2024_006_0).

### Summary of the three original clinical trials

For detailed methods of Trials A, B, and C, please refer to the respective original publications [18–20].

#### Trial A.

This trial was a phase 3, multicenter, randomized, double-blind, placebo-controlled, parallel-group trial conducted at 34 facilities. HD patients whose serum phosphorus levels were within the target range while on a stable dose of PBs were pre-enrolled. They were formally enrolled if their serum phosphorus levels increased by ≥1.0 mg/dL and reached 6.1–9.9 mg/dL during the PB washout period. Enrolled patients received either tenapanor or placebo twice daily for 8 weeks. Tenapanor treatment was initiated at 5 mg twice daily and titrated stepwise to 10, 20, or 30 mg twice daily at intervals of at least 2 weeks, according to serum phosphorus levels and the occurrence of GI symptoms.

#### Trial B.

This trial was a phase 3, randomized, double-blind, placebo-controlled, parallel-group trial conducted at 40 facilities. The trial included a screening period, a run-in period, and an 8-week double-blind treatment period. HD patients with serum phosphorus levels ≥6.1 mg/dL and <10.0 mg/dL at screening, despite treatment with one or more PBs, were enrolled. During the 8-week treatment period, serum phosphorus levels were masked; however, the central laboratory issued alerts based on predefined thresholds: treatment was to be discontinued if the serum phosphorus level was ≥ 10.0 mg/dL; the dose was to be increased if it was ≥ 6.1 mg/dL; the dose could be increased if it was between 4.5 and 6.0 mg/dL; the dose was to be maintained if it was between 3.5 and 4.5 mg/dL; and the dose was to be decreased if it was < 3.5 mg/dL. After 2 weeks of tenapanor administration, the dose was escalated stepwise using a 5 mg up-titration scheme (i.e., 5 mg → 10 mg → 20 mg → 30 mg twice daily), with a minimum 2-week interval between dose adjustments. During the study period, changes to the dose, formulation, type, or content of concomitant PBs were generally not permitted.

#### Trial C.

This trial was a phase 3, open-label, multicenter, single-arm clinical trial conducted at 20 facilities. The trial consisted of a screening period, a PB washout period, and a 16-week treatment period. The main inclusion criteria were as follows: patients undergoing peritoneal dialysis (PD) for ≥12 weeks; receiving PBs at a fixed dosage; having serum phosphorus levels of 3.5–7.0 mg/dL before PB washout; and showing an increase in serum phosphorus to 6.1–10.0 mg/dL at 1 or 2 weeks after PB discontinuation. The starting dose of tenapanor was 5 mg, which was titrated stepwise to 10, 20, or 30 mg per dose. Dose adjustments (i.e., increase, reduction, or discontinuation) were performed every 2 weeks according to the same decision criteria as in Trial B.

### Endpoints

As efficacy endpoints, changes in the tenapanor dosage and serum phosphorus levels were assessed. These data were collected weekly from Week 0 to Week 8. Based on the final tenapanor dosage at Week 8, patients were stratified into a low-dose group (5 and 10 mg twice daily) and a high-dose group (20 and 30 mg twice daily). If the tenapanor dose data at Week 8 are missing due to interruption of medication or discontinuation of patient, the most recent dose data prior to Week 8 was used.

As safety endpoints, all adverse drug reactions (ADRs) related to the GI system were assessed. ADRs reported during the trials were classified using the Medical Dictionary for Regulatory Activities (MedDRA) version 24.1.

### Dataset

Efficacy endpoints were evaluated in the modified intention-to-treat population, which included all patients who did not fall into either of the following categories: those who did not receive tenapanor and those for whom serum phosphorus levels were not measured after the start of tenapanor administration. Safety endpoints were assessed in the safety analysis set, comprising all patients who received at least one dose of tenapanor. Baseline (Week 0) predictors of the final tenapanor dose were evaluated using data from the modified intention-to-treat population.

### Statistical methods

#### Descriptive statistics and group comparisons.

Frequencies and percentages were calculated for nominal and ordinal variables in the low-dose and high-dose groups. For continuous variables, means and standard deviations (SDs) or medians and interquartile ranges were determined, depending on data distribution, for all patients as well as for the two dose subgroups.

The following patient characteristics at baseline were summarized: sex; age; weight; body mass index (BMI); presence of primary kidney disease; dialysis duration; dialysis modality; Kt/V urea; serum levels of phosphorus, corrected calcium, intact parathyroid hormone, tartrate-resistant acid phosphatase 5b (TRACP-5b), bone-specific alkaline phosphatase, osteocalcin, and procollagen type 1 N-terminal propeptide; number of patients prescribed each PB; number of PB types; use of concomitant medications including laxatives, antidiarrheals, and acid-suppressing agents (proton pump inhibitors and H2-receptor antagonists); and use of oral and intravenous vitamin D receptor activators. Additionally, the Bristol Stool Form Scale (BSFS) score and weekly stool frequency were recorded. It should be noted that in Trials A and C, where PBs were washed out, PB prescriptions refer to those used prior to the washout period rather than at baseline.

#### Serum phosphorus levels and target range achievement.

For the efficacy analysis, the mean (± SD) of serum phosphorus levels was calculated at 1-week intervals from Week 0 to Week 8 for each subgroup. The mixed model for repeated measures (MMRM) was used to compare the changes from baseline in serum phosphorus levels in the tenapanor and placebo groups. The proportion of patients who achieved the target serum phosphorus range (3.5–6.0 mg/dL) recommended by the Japanese Society for Dialysis Therapy [22] was also calculated weekly from Week 0 to Week 8, based on the number of patients whose serum phosphorus levels were above, within, or below the target range. Differences in the proportion of patients achieving the target serum phosphorus range were identified using Fisher’s exact test.

#### Safety analysis.

For the safety analysis, the incidence rates of all ADRs related to the GI tract were calculated for each subgroup. Differences in the incidence rates of GI-related ADRs were identified using Fisher’s exact test.

#### Analysis of baseline predictors of final tenapanor dosage.

Patients were stratified into low-dose and high-dose groups according to their tenapanor dose. Univariate and multivariate logistic analyses were conducted to identify baseline characteristics associated with the requirement for higher tenapanor doses, using variables obtained at baseline. The variables included in the univariate and multivariable analyses were prespecified based on clinical relevance. In the univariate analysis, the following baseline variables were evaluated: sex, age (per 10-year increase), BMI (per 1 kg/m² increase), presence of diabetic nephropathy, dialysis duration (per 1-year increase), dialysis modality (HD, hemodiafiltration [HDF], and PD), use of PBs (calcium carbonate, sevelamer hydrochloride, lanthanum carbonate, bixalomer, ferric citrate hydrate, and sucroferric oxyhydroxide), number of PB types (per additional type), serum phosphorus (per 1 mg/dL increase), corrected calcium (per 1 mg/dL increase), intact parathyroid hormone (per 100 pg/mL increase), TRACP-5b (per 100 mU/dL increase), use of concomitant medications (laxatives, acid-suppressing agents, and vitamin D receptor activators), BSFS score, and weekly stool frequency (per one increase). In the multivariable analysis, the following prespecified variables were included: sex, age, BMI, presence of diabetic nephropathy, dialysis duration, use of sevelamer hydrochloride, number of PB types, serum phosphorus, intact parathyroid hormone, TRACP-5b, use of concomitant medications (laxatives, acid-suppressing agents, and vitamin D receptor activators), BSFS score, and weekly stool frequency.

To identify baseline predictive factors, a logistic regression model was applied to evaluate the association between patient characteristics at baseline and the likelihood of belonging to either the low-dose group (5 and 10 mg/dose) or the high-dose group (20 and 30 mg/dose) at Week 8. The variables included in the model were selected based on statistical significance in univariate analysis, clinical relevance, sample size considerations, and assessment of multicollinearity.

All statistical analyses were performed by an external specialized organization, independent of the funder. All statistical analyses were performed using SAS version 9.4 (SAS Institute, Cary, NC, USA), and a p-value of <0.05 was considered statistically significant.

## Results

### Patient analysis sets

In the present study, a total of 212 patients who received tenapanor at Week 8 were stratified into a low-dose group (5 and 10 mg/dose; n = 127) and a high-dose group (20 and 30 mg/dose; n = 85) for the efficacy analysis. These patients comprised 81 from Trial A, 80 from Trial B, and 51 from Trial C. In addition, 218 patients were included in the safety analysis set to evaluate GI-related ADRs.

### Patient characteristics

At baseline, 138 patients (65.1%) were male, with a mean age of 63.2 years, and 68 patients (32.1%) had diabetic nephropathy (Table 1). The average duration of dialysis was 93.1 months. Regarding dialysis modality, 107 patients (50.5%) were receiving HDF, 54 (25.5%) HD, and 51 (24.1%) PD. The mean serum phosphorus and corrected calcium levels were 7.39 and 8.88 mg/dL, respectively. As for PB prescriptions, lanthanum carbonate was the most commonly used (59.0%), followed by calcium carbonate (48.1%), iron-containing formulations (38.7%), and polymer-based formulations (19.8%). Notably, more than half of the patients (54.7%) were prescribed two or more types of PBs. Laxatives were prescribed to 36.8% of patients. The mean baseline BSFS score was 4.04, and the average stool frequency was 8.24 times per week.

**Table 1:** Patient characteristics at baseline in all patients, and in the low-dose and high-dose groups as defined at Week 8.

Characteristics^a^	Total (n = 212)	Low-dose group (n = 127)	High-dose group (n = 85)
**Sex (male)**	138 (65.1)	82 (64.6)	56 (65.9)
**Age, years**	63.2 ± 10.9	63.7 ± 11.0	62.3 ± 10.8
**Weight, kg**	64.4 ± 13.0	63.6 ± 12.7	65.7 ± 13.5
**BMI, kg/m** ^**2**^	24.3 ± 4.2	24.1 ± 4.0	24.6 ± 4.5
**Primary kidney disease**			
** CGN**	75 (35.4)	37 (29.1)	38 (44.7)
** DN**	68 (32.1)	37 (29.1)	31 (36.5)
** Nephrosclerosis**	31 (14.6)	23 (18.1)	8 (9.4)
** Other**	38 (17.9)	30 (23.6)	8 (9.4)
**Dialysis duration, months**	93.1 ± 83.5	87.9 ± 82.0	100.9 ± 85.6
**Dialysis modality**			
** HDF**	107 (50.5)	70 (55.1)	37 (43.5)
** HD**	54 (25.5)	28 (22.0)	26 (30.6)
** PD**	51 (24.1)	29 (22.8)	22 (25.9)
**Kt/V urea**	1.55 ± 0.25	1.55 ± 0.22	1.55 ± 0.29
**Serum chemistry**			
** Phosphorus, mg/dL**	7.39 ± 1.30	7.24 ± 1.37	7.62 ± 1.17
** cCa, mg/dL**	8.88 ± 0.66	8.87 ± 0.68	8.88 ± 0.64
** i-PTH, pg/mL**	200 (120–309)	196 (113–291)	208 (136–320)
** TRACP-5b, mU/dL**	583.6 ± 350.3	532.4 ± 312.9	660.1 ± 389.3
** BAP, μg/L**	12.6 ± 5.9	12.4 ± 6.1	12.8 ± 5.7
** Osteocalcin, ng/mL**	176.4 ± 121.0	163.2 ± 109.1	196.1 ± 135.1
** P1NP, μg/L**	267.3 ± 193.8	265.7 ± 192.2	269.5 ± 197.3
**PBs** ^**b**^			
** Lanthanum carbonate**	125 (59.0)	74 (58.3)	51 (60.0)
** Calcium carbonate**	102 (48.1)	60 (47.2)	42 (49.4)
** Ferric citrate hydrate**	52 (24.5)	26 (20.5)	26 (30.6)
** Sucroferric oxyhydroxide**	30 (14.2)	18 (14.2)	12 (14.1)
** Sevelamer hydrochloride**	21 (9.9)	8 (6.3)	13 (15.3)
** Bixalomer**	21 (9.9)	12 (9.4)	9 (10.6)
**No. of PB types**			
** 1**	96 (45.3)	65 (51.2)	31 (36.5)
** ≥2**	116 (54.7)	62 (48.8)	54 (63.5)
**Concomitant medications**			
** Laxatives**	78 (36.8)	39 (30.7)	39 (45.9)
** Antidiarrheals**	6 (2.8)	3 (2.4)	3 (3.5)
** Acid-suppressing agents**	122 (57.5)	73 (57.5)	49 (57.6)
** Proton pump inhibitors**	110 (90.2)	66 (90.4)	44 (89.8)
** H2-receptor antagonists**	12 (9.8)	7 (9.6)	5 (10.2)
** VDRAs**	173 (81.6)	102 (80.3)	71 (83.5)
** Intravenous VDRAs**	81 (46.8)	53 (52.0)	28 (39.4)
**BSFS (score)**	4.04 ± 0.89	4.15 ± 0.87	3.88 ± 0.89
**Stool frequency, times/week**	8.24 ± 3.60	8.17 ± 3.76	8.35 ± 3.37

BAP, bone-specific alkaline phosphatase; BMI, body mass index; BSFS, Bristol Stool Form Scale; cCa, corrected calcium; CGN, chronic glomerulonephritis; DN, diabetic nephropathy; HD, hemodialysis; HDF, hemodiafiltration; i-PTH, intact parathyroid hormone; P1NP, procollagen type 1 N-terminal propeptide; PB, phosphate binder; PD, peritoneal dialysis; TRACP-5b, tartrate-resistant acid phosphatase 5b; VDRA, vitamin D receptor activator.

^a^Data are n (%), mean ± standard deviation, or median (lower quartile to upper quartile).

^b^In Trials A and C, where PBs were washed out, PB prescriptions refer to those used prior to the washout period rather than at baseline.

Univariate analysis revealed that, compared with the low-dose group, the high-dose group had significantly higher serum phosphorus and TRACP-5b levels, a greater proportion of patients prescribed sevelamer hydrochloride, laxatives, and multiple types of PBs, and significantly lower BSFS scores (Table 2).

**Table 2:** Univariate logistic analysis comparing the low-dose and high-dose groups as defined at Week 8, and multivariate logistic analysis to identify predictors of higher tenapanor dose requirement.

Explanatory variable	Univariate analysis		Multivariate analysis	
	OR (95% CI)	p-value	OR (95% CI)	p-value
**Sex (male)**	1.060 [0.595–1.887]	0.844	1.249 [0.642–2.429]	0.513
**Age (10 years)**	0.890 [0.691–1.144]	0.362	0.855 [0.632–1.156]	0.308
**BMI (kg/m**^**2**^)	1.031 [0.966–1.101]	0.358	1.069 [0.983–1.163]	0.120
**DN (existence)**	1.396 [0.778–2.505]	0.263	1.474 [0.723–3.008]	0.286
**Dialysis duration (years)**	1.022 [0.983–1.063]	0.268	1.028 [0.975–1.083]	0.308
**Dialysis modality**				
** HDF**	0.697 [0.352–1.379]	0.299	–	–
** HD**	1.224 [0.567–2.642]	0.607	–	–
**Serum chemistry**				
** Phosphorus (per 1 mg/dL)**	1.257 [1.015–1.558]	0.036	1.223 [0.964–1.553]	0.098
** cCa (per 1 mg/dL)**	1.011 [0.667–1.532]	0.959	–	–
** i-PTH (per 100 pg/mL)**	1.078 [0.869–1.337]	0.494	0.914 [0.692–1.207]	0.527
** TRACP-5b (per 100 mU/dL)**	1.111 [1.025–1.204]	0.011	1.121 [1.005–1.249]	0.040
**PBs**				
** Lanthanum carbonate (user)**	1.074 [0.614–1.879]	0.802	–	–
** Calcium carbonate (user)**	1.091 [0.629–1.890]	0.757	–	–
** Ferric citrate hydrate (user)**	1.712 [0.910–3.219]	0.095	–	–
** Sucroferric oxyhydroxide (user)**	0.995 [0.453–2.190]	0.991	–	–
** Sevelamer hydrochloride (user)**	2.686 [1.062–6.794]	0.037	2.475 [0.813–7.537]	0.111
** Bixalomer (user)**	1.135 [0.456–2.824]	0.786	–	–
**Number of PB types (per additional type)**	1.674 [1.111–2.523]	0.014	1.251 [0.772–2.029]	0.363
**Concomitant medications**				
** Laxatives (user)**	1.913 [1.083–3.381]	0.026	1.724 [0.895–3.318]	0.103
** Acid-suppressing agents (user)**	1.007 [0.578–1.755]	0.981	0.726 [0.376–1.402]	0.340
** Intravenous VDRAs (user)**	0.686 [0.386–1.217]	0.198	0.525 [0.254–1.085]	0.082
**BSFS (score)**	0.709 [0.516–0.975]	0.034	0.667 [0.468–0.951]	0.025
**Stool frequency (times/week)**	1.014 [0.940–1.095]	0.713	1.020 [0.935–1.112]	0.659

BMI, body mass index; BSFS; Bristol Stool Form Scale; cCa, corrected calcium; CI, confidence interval; DN, diabetic nephropathy; HD, hemodialysis; HDF, hemodiafiltration; i-PTH, intact parathyroid hormone; OR, odds ratio; PB, phosphate binder; TRACP-5b, tartrate-resistant acid phosphatase 5b; VDRA; vitamin D receptor activator.

* p < 0.05.

### Changes in dose and serum phosphorus levels

**Fig 1:**
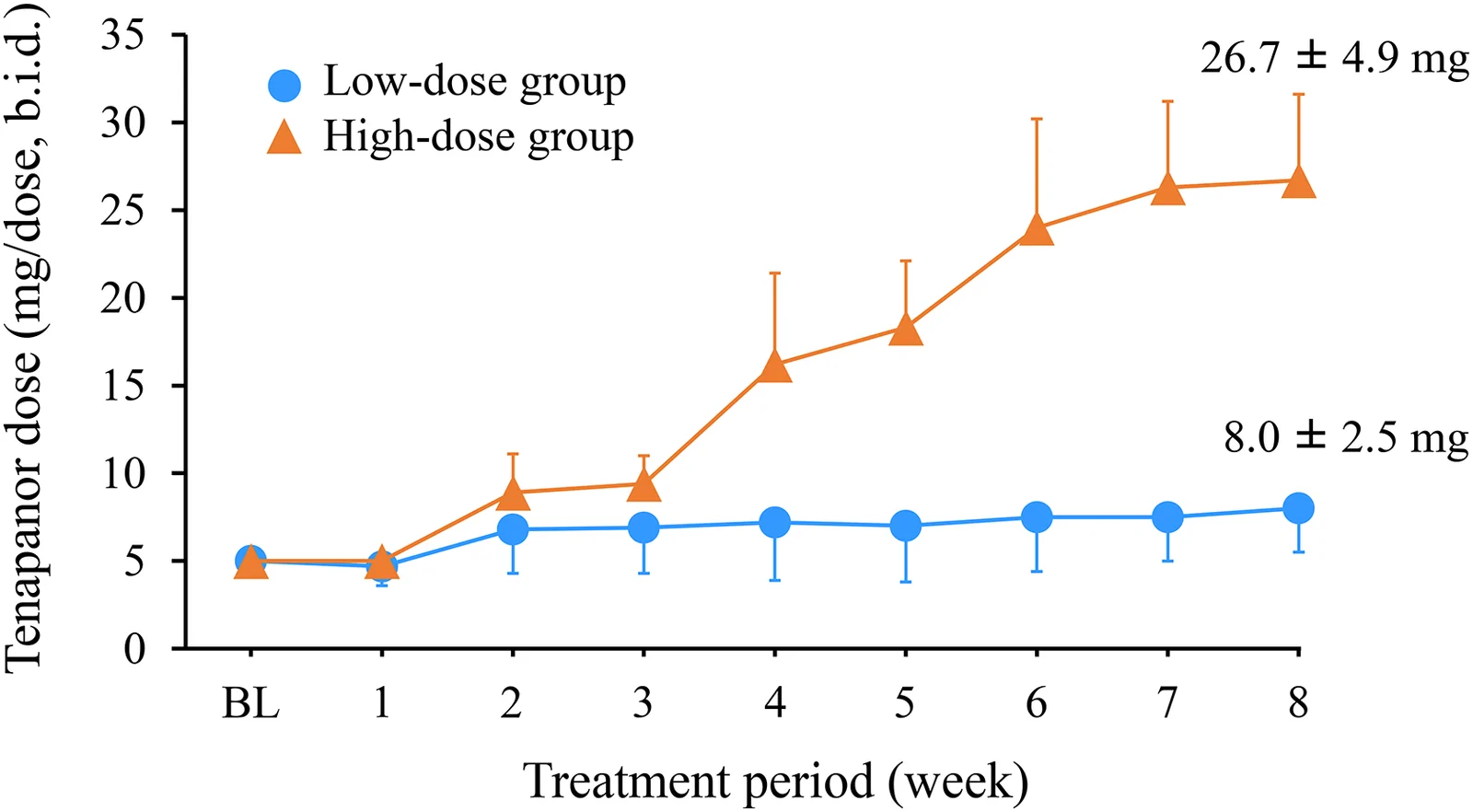
Mean changes in tenapanor dosage over time in low-dose and high-dose groups. b.i.d., twice daily; BL, baseline. Error bars represent standard deviation.

All patients initiated tenapanor treatment at a dose of 5 mg twice daily. After 8 weeks of treatment, patients were retrospectively stratified into a low-dose group (5 and 10 mg/dose; n = 127) and a high-dose group (20 and 30 mg/dose; n = 85) according to their final dose. In the high-dose group, the tenapanor dose was gradually increased, reaching 26.7 ± 4.9 mg twice daily by Week 8 (Fig 1). By contrast, in the low-dose group, the dose increased only slightly, reaching 8.0 ± 2.5 mg twice daily at Week 8.

**Fig 2:**
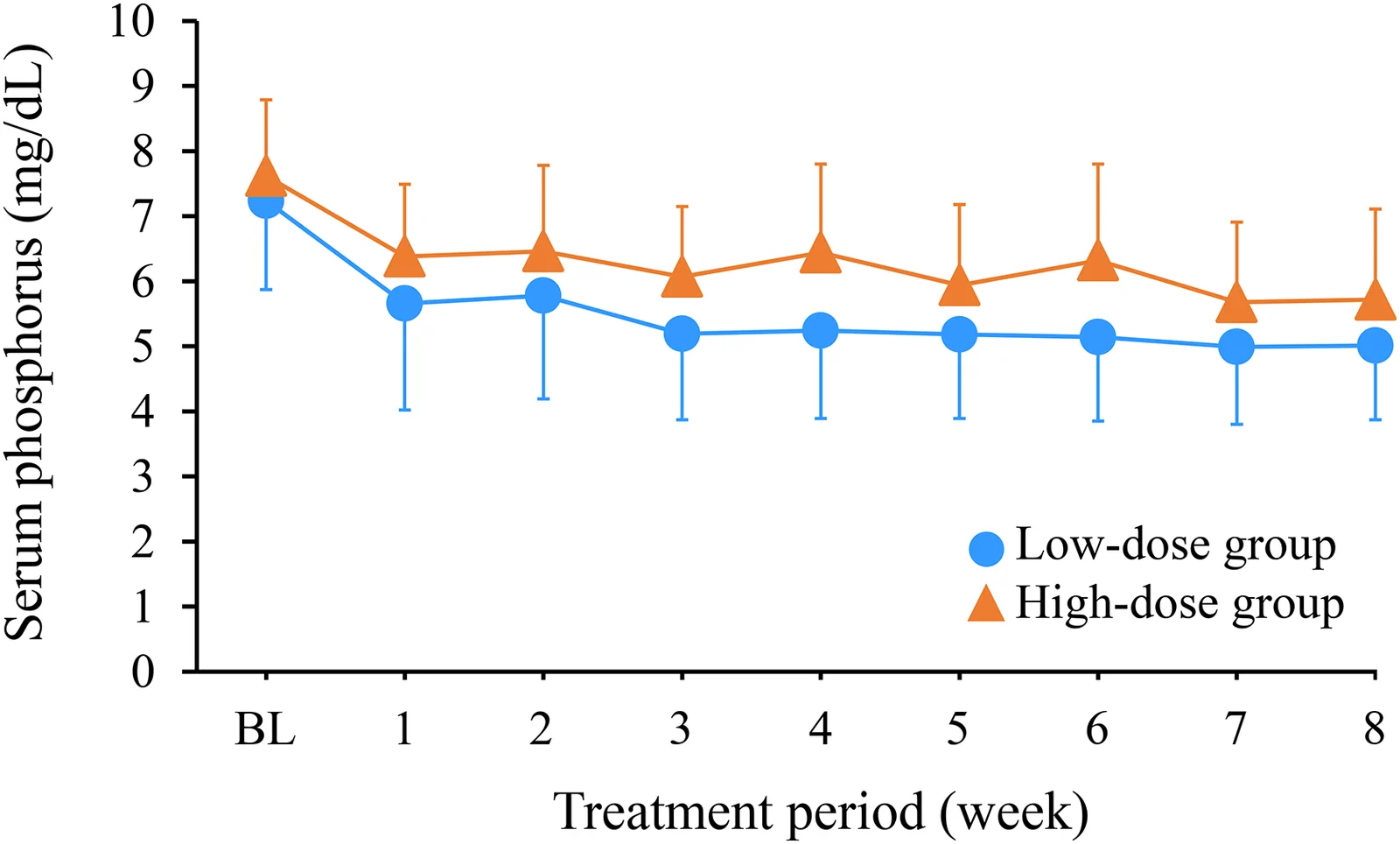
Mean changes in serum phosphorus levels over time in the low-dose and high-dose groups. BL, baseline. Error bars represent standard deviation.

After initiation of tenapanor treatment, serum phosphorus levels gradually declined in both groups over the 8-week period (Fig 2). Throughout the observation period, serum phosphorus levels remained lower in the low-dose group than in the high-dose group; however, the magnitude of change from baseline did not differ significantly between groups (p = 0.456, MMRM).

**Fig 3:**
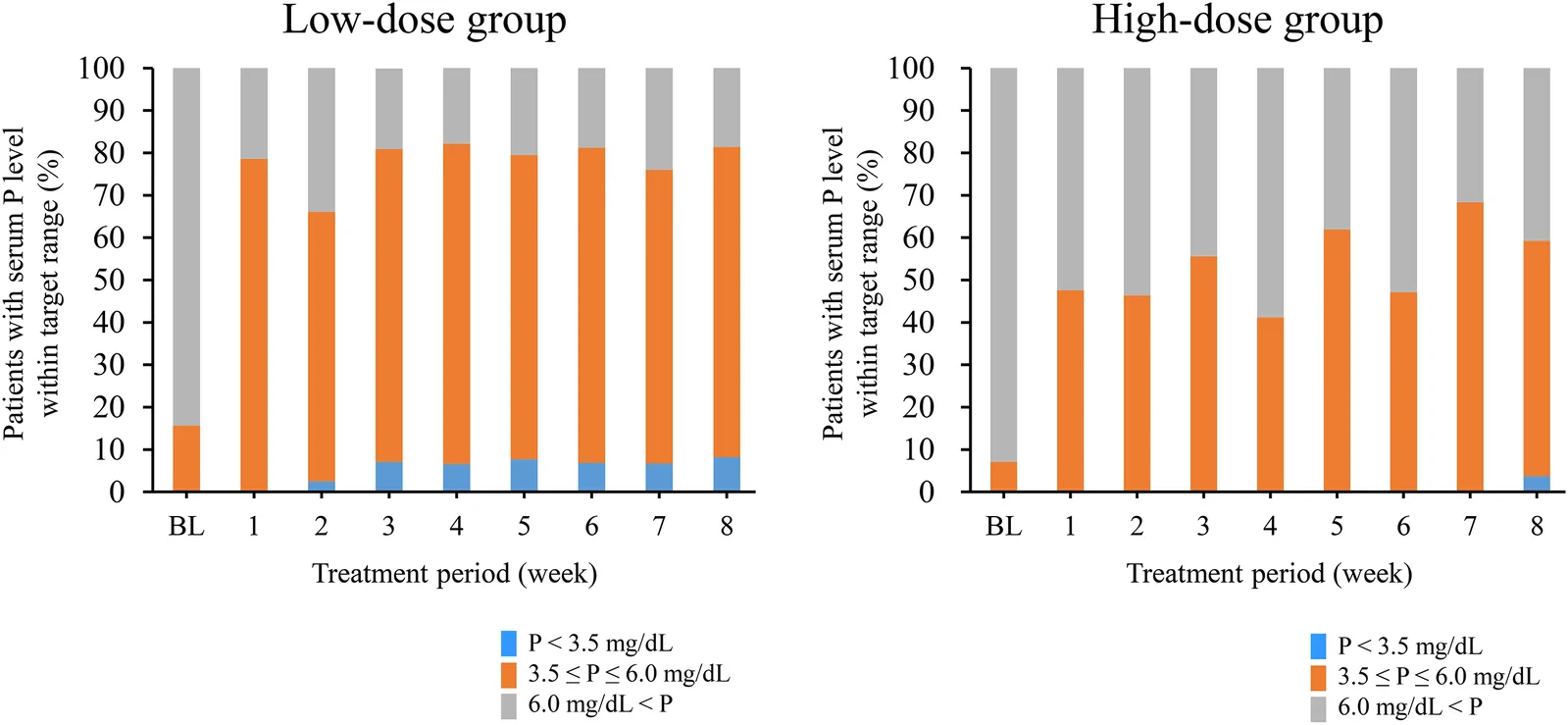
Proportion of patients who achieved the target serum phosphorus range (3.5–6.0 mg/dL) recommended by the Japanese Society for Dialysis Therapy in the low-dose and high-dose groups over time. BL, baseline; P, phosphorus.

The proportion of patients who achieved the target serum phosphorus range (3.5–6.0 mg/dL) recommended by the Japanese Society for Dialysis Therapy is shown in Fig 3. In both groups, the number of patients within the target range increased after 1 week of tenapanor initiation and was maintained through Week 8. However, the low-dose group tended to have a higher proportion of patients within the target range, with a statistically significant difference observed at Week 8 (p < 0.05, Fisher’s exact test).

### Predictors of higher tenapanor dose requirement

Multivariate analysis identified serum TRACP-5b level as a significant independent positive predictor, and BSFS score as a significant independent negative predictor, for requiring higher doses of tenapanor (Table 2).

### Safety

For detailed information on tenapanor-related adverse events observed in Trials A, B, and C, please refer to the respective original publications [18–20]. Table 3 presents the incidence rates of GI-related ADRs occurring at a frequency of 5% or higher in the low-dose and high-dose groups. Patients in both groups had a high incidence of diarrhea, while soft stools were reported in only a small number of cases. No statistically significant differences in the incidence rates of GI-related ADRs were observed between the groups.

**Table 3:** Incidence of gastrointestinal-related adverse drug reactions occurring at a frequency of 5% or higher in the low-dose and high-dose groups in the safety analysis set^a^.

Adverse event	Low-dose group (n = 133)	High-dose group (n = 85)	p-value^b^
**Diarrhea**	95 (71.4)	57 (67.1)	0.546
**Soft stools**	7 (5.3)	5 (5.9)	1.000

^a^Data are n (%).

^b^Fisher’s exact test.

## Discussion

In this pooled analysis of data from three clinical trials, multivariate analysis identified higher serum TRACP-5b levels and lower BSFS scores as independent predictors of requiring higher tenapanor doses. The clinical significance of these findings is discussed below.

TRACP-5b is a reliable marker of bone resorption in dialysis patients [23]. Elevated levels reflect increased osteoclast number and activity, often seen in high-turnover bone disease, particularly secondary hyperparathyroidism. Notably, in the present study, parathyroid hormone (a bone formation marker) showed no univariate association with tenapanor dose, whereas TRACP-5b (a bone resorption marker) did. In addition, baseline levels of bone-specific alkaline phosphatase, reflecting osteoblast activity, and procollagen type 1 N-terminal propeptide, reflecting collagen production, were largely comparable between the low-dose and high-dose groups. Taken together, these findings support the notion that bone resorption, rather than bone formation, is more closely associated with tenapanor dose requirements. Mechanistically, approximately 85% of total body phosphorus is stored in bone as hydroxyapatite, and osteoclast-mediated resorption releases P into the circulation. Calcimimetics lower serum phosphorus levels by reducing this bone-derived P flux via suppression of parathyroid hormone [24]. By contrast, tenapanor acts by inhibiting intestinal P absorption and does not directly affect bone resorption. Therefore, patients with greater P release from bone, reflected by higher TRACP-5b levels, may require higher tenapanor doses to achieve target serum phosphorus levels. This interpretation is supported by the observation that serum phosphorus levels remained higher in the high-dose group than in the low-dose group throughout the study period. Overall, these results suggest that bone-derived P flux may influence tenapanor dose requirements more than bone formation markers. Clinically, TRACP-5b measurement could help identify dialysis patients needing more intensive serum phosphorus-lowering strategies, including higher tenapanor doses or combination therapy with calcimimetics. Prospective studies integrating bone turnover markers with phosphorus kinetics are warranted.

The BSFS classifies stools into seven types according to form and consistency: Types 1 and 2 indicate constipation (hard lumps or lumpy sausage shapes); Types 3 and 4 represent normal stools (cracked sausages to smooth, soft shapes); Type 5 consists of soft blobs with clear-cut edges; and Types 6 and 7 indicate diarrhea (mushy to watery stools) [25,26]. In our study, multivariate logistic regression analysis showed that patients with lower BSFS scores, reflecting a tendency toward constipation, were more likely to require higher tenapanor doses. Tenapanor inhibits the sodium/hydrogen exchanger isoform 3, causing Na⁺ to accumulate in the intestinal lumen and increasing luminal osmotic pressure. This osmotic gradient drives water movement from the interstitium into the lumen, resulting in softer stools and diarrhea, which are well-recognized adverse effects of the drug. In rats, a single oral dose of tenapanor increased both ileal luminal water content and fecal Na⁺ excretion, consistent with these clinical observations [27]. In the present study, univariate analysis showed that laxatives and sevelamer hydrochloride were prescribed to a significantly higher proportion of patients in the high-dose group, with the latter known to cause constipation as an adverse effect [3–5]. Moreover, no significant difference was observed in the incidence of GI-related ADRs between the low-dose and high-dose groups. These findings are consistent with a recent report indicating that patients who continue tenapanor therapy are more likely to be habitual laxative users than those who discontinue it [28], as well as with result that tenapanor prescriptions are associated with reduced laxative use [29]. Therefore, in patients prone to constipation, titration of tenapanor may be achieved more readily due to its osmotic, laxative-like effect.

The present study has several limitations, as it is a pooled analysis of data from three clinical trials with differing designs. First, it included both tenapanor monotherapy after PB washout and combination therapy with PBs. In the combination therapy group, serum phosphorus levels and GI-related ADRs may have been influenced by the type and dose of PBs, potentially affecting tenapanor dose adjustments. Separate analyses of the monotherapy and combination therapy trials were not feasible due to the small number of subjects. Nonetheless, the findings remain clinically relevant, as tenapanor is used both as monotherapy and in combination with PBs in real-world practice. The three trials differed in several aspects, which may introduce some degree of heterogeneity; however, the objective of this study was not to compare treatment effects across trials but to exploratorily identify baseline characteristics associated with achieving higher doses. Second, the study included 162 HD patients and 52 PD patients. However, when treated with tenapanor monotherapy, the dose, frequency of diarrhea, and frequency of soft stools were comparable between the two groups [18,20]. Third, although Trial C included a total of 16 weeks of tenapanor treatment, the primary endpoint was the change in serum phosphorus levels after 8 weeks; therefore, only data up to Week 8 were used. Because the mean tenapanor dose at Week 16 (16.8 mg) was similar to that at Week 8 (16.4 mg) [20], it is likely that dose escalation had been essentially completed by Week 8. Fourth, the sample size of the present study was relatively small for a pooled analysis. However, a key strength is that it used data from high-quality, multicenter clinical trials conducted under Good Clinical Practice and in strict compliance with the trial protocols, allowing analysis of a patient population with high medication adherence who followed the prescribed dosage and administration accurately. Finally, because dose titration was based on serum phosphorus levels and tolerability, and group classification was based on the final dose, the possibility of circular inference cannot be completely excluded, although baseline serum phosphorus was included in the multivariable analysis. Furthermore, the relatively large number of explanatory variables compared with the number of patients in the high-dose group raises the possibility of overfitting.

In conclusion, patients requiring higher doses of tenapanor are characterized by either increased bone resorption or a propensity for constipation. Increased bone resorption may attenuate the drug’s phosphorus-lowering effect by contributing to a greater release of P from bone, whereas constipation may facilitate more aggressive dose titration owing to the drug’s stool-softening properties. Accordingly, incorporating assessments of bone turnover and bowel habits into routine clinical evaluations may help guide individualized tenapanor dosing. Further large-scale prospective studies in real-world clinical settings are warranted to validate these findings.
