# Supplementary material for: Patient characteristics associated with the need for high doses of tenapanor hydrochloride in dialysis patients with hyperphosphatemia: A pooled analysis of three clinical trials
Source: PLoS One. 2026 Aug 28;21(8):e0356873. doi: 10.1371/journal.pone.0356873 (PMC13524331; doi:10.1371/journal.pone.0356873)
Supplement: Supplementary file 1 [file pone.0356873.s002.doc]

**Response to Editor:**

1. Please ensure that your manuscript meets PLOS ONE's style requirements, including those for file naming. The PLOS ONE style templates can be found at <https://journals.plos.org/plosone/s/file?id=wjVg/PLOSOne_formatting_sample_main_body.pdf> and [https://journals.plos.org/plosone/s/file?id=ba62/PLOSOne_formatting_sample_title_authors_affiliations.pdf](https://jpn01.safelinks.protection.outlook.com/?url=https%3A%2F%2Ftrack.editorialmanager.com%2FCL0%2Fhttps%3A%252F%252Fjournals.plos.org%252Fplosone%252Fs%252Ffile%253Fid%3Dba62%252FPLOSOne_formatting_sample_title_authors_affiliations.pdf%2F1%2F010f019d948d2345-4c280e4d-e350-4a95-83d8-13705bc28b6d-000000%2F-AkhAs14a0UQ0uDXCaOMAXNzTkZZqIhCLS2jnzjdwyI%3D258&data=05|02|shin.tokunaga.jg@kyowakirin.com|fcb83e0ec22e4baa4b2b08de9b721f06|34db9a5d91ba486ebb82d37d2ee1d84c|0|0|639119112016286510|Unknown|TWFpbGZsb3d8eyJFbXB0eU1hcGkiOnRydWUsIlYiOiIwLjAuMDAwMCIsIlAiOiJXaW4zMiIsIkFOIjoiTWFpbCIsIldUIjoyfQ%3D%3D|0|||&sdata=CX2zffSNeWcUsDfI8sdDEf0%2FaCbuumoHGNVZnPuxN%2FY%3D&reserved=0)

***Response:*** *Thank you for your comment. In preparation for resubmission, we revised the manuscript text in accordance with the PLOS ONE style template.*

2. We note that the grant information you provided in the ‘Funding Information’ and ‘Financial Disclosure’ sections do not match.

When you resubmit, please ensure that you provide the correct grant numbers for the awards you received for your study in the ‘Funding Information’ section.

***Response:*** *Thank you for your comment. We have reviewed and revised the statements in the “Funding Information” and “Financial Disclosure” sections.*

3. Thank you for stating the following in the Competing Interests section:

“Nobuo Nagano received consultancies from Kyowa Kirin Co., Ltd.; honoraria from Kyowa Kirin Co., Ltd.; Kissei Pharmaceutical Co., Ltd.; Toa Shinyaku Co., Ltd.; Nobelpharma Co., Ltd.; Torii Pharmaceutical Co.,Ltd.; and Ono Pharmaceutical Co., Ltd.

Shin Tokunaga and Shinji Asada are employees of Kyowa Kirin Co., Ltd.

Masafumi Fukagawa received grants from Kyowa Kirin Co., Ltd. to his institution; consulting fees and honoraria from Sanwa Kagaku Kenkyusho Co., Ltd.; Ono Pharmaceutical Co., Ltd.; Kyowa Kirin Co., Ltd.; Bayer Yakuhin, Ltd.; Kissei Pharmaceutical Co., Ltd.; and Torii Pharmaceutical Co., Ltd.

Tadao Akizawa received consulting fees from Kyowa Kirin Co., Ltd.; Kissei Pharmaceutical Co., Ltd.; Torii Pharmaceutical Co., Ltd.; and Sanwa Kagaku Kenkyusho Co., Ltd.; honoraria from Kyowa Kirin Co., Ltd.; Kissei Pharmaceutical Co., Ltd.; Ono Pharmaceutical Co., Ltd.; Torii Pharmaceutical Co., Ltd.; and Sanwa Kagaku Kenkyusho Co., Ltd.; support for travel fee from Kyowa Kirin Co., Ltd.”

We note that one or more of the authors are employed by a commercial company: Kyowa Kirin Co., Ltd.; Kissei Pharmaceutical Co., Ltd.; Toa Shinyaku Co., Ltd.; Nobelpharma Co., Ltd.; Torii Pharmaceutical Co.,Ltd.; and Ono Pharmaceutical Co., Ltd. And Sanwa Kagaku Kenkyusho Co., Ltd.;

1. Please provide an amended Funding Statement declaring this commercial affiliation, as well as a statement regarding the Role of Funders in your study. If the funding organization did not play a role in the study design, data collection and analysis, decision to publish, or preparation of the manuscript and only provided financial support in the form of authors' salaries and/or research materials, please review your statements relating to the author contributions, and ensure you have specifically and accurately indicated the role(s) that these authors had in your study. You can update author roles in the Author Contributions section of the online submission form.

Please also include the following statement within your amended Funding Statement.

“The funder provided support in the form of salaries for authors [insert relevant initials], but did not have any additional role in the study design, data collection and analysis, decision to publish, or preparation of the manuscript. The specific roles of these authors are articulated in the ‘author contributions’ section.”

If your commercial affiliation did play a role in your study, please state and explain this role within your updated Funding Statement.

***Response:*** *Thank you for your comment. We have reviewed the requirements regarding commercial affiliations and the role of funders and have revised the submission information accordingly.*

*In the revised Funding Statement, we have clarified that the funder provided support in the form of salaries for ST and AS and was involved in the study design and conduct of the study, as well as in the decision to submit the manuscript. Statistical analyses were performed by an external specialized organization independent of the funder. The specific roles of these authors are articulated in the ‘author contributions’ section.*

2. Please also provide an updated Competing Interests Statement declaring this commercial affiliation along with any other relevant declarations relating to employment, consultancy, patents, products in development, or marketed products, etc.

Within your Competing Interests Statement, please confirm that this commercial affiliation does not alter your adherence to all PLOS ONE policies on sharing data and materials by including the following statement: "This does not alter our adherence to  PLOS ONE policies on sharing data and materials.” (as detailed online in our guide for authors <http://journals.plos.org/plosone/s/competing-interests>) . If this adherence statement is not accurate and  there are restrictions on sharing of data and/or materials, please state these. Please note that we cannot proceed with consideration of your article until this information has been declared.

Please include both an updated Funding Statement and Competing Interests Statement in your cover letter. We will change the online submission form on your behalf.

***Response:*** *Thank you for your comment. We have reviewed the requirements for the declaration of competing interests and prepared an updated Competing Interests Statement. We have also included the following required wording in the Competing Interests Statement:*

*“This does not alter our adherence to PLOS ONE policies on sharing data and materials.”*

4. Your ethics statement should only appear in the Methods section of your manuscript. If your ethics statement is written in any section besides the Methods, please move it to the Methods section and delete it from any other section. Please ensure that your ethics statement is included in your manuscript, as the ethics statement entered into the online submission form will not be published alongside your manuscript.

***Response:*** *Thank you for your comment. We have reviewed the ethics statement. The content that had been placed outside the Methods section was moved into the Methods section of the manuscript and revised accordingly.*

***Lines 103-107:***

*Ethical approval of each clinical trial [18-20] was obtained from the ethics committees of all 94 participating institutions, and written informed consent was obtained from each patient prior to participation. This pooled post hoc analysis was approved by the ethics committee of Kyowa Kirin Co., Ltd (approval number: MA2024_006_0).*

5. If the reviewer comments include a recommendation to cite specific previously published works, please review and evaluate these publications to determine whether they are relevant and should be cited. There is no requirement to cite these works unless the editor has indicated otherwise.

***Response:*** *Thank you for your comment. We have reviewed the reviewer comments and found no explicit recommendations to cite specific previously published works.*

**Response to Reviewer 1:**

1. This study pooled data from three clinical trials with heterogeneous designs. However, there is no evaluation or discussion on its effect on the results or conclusions.

***Response:*** *Thank you for this important comment. We acknowledge that the three phase 3 clinical trials included in this study differ in certain aspects, such as dialysis modality (HD/PD) and the use or washout of phosphate binders, resulting in some degree of heterogeneity across the trials. However, the objective of this study was not to compare treatment effects across trials, but rather to exploratorily identify baseline characteristics common to patients who ultimately reached high doses within clinical trials where dose escalation was conducted stepwise according to protocol.*

*In addition, all trials were conducted in Japanese patients undergoing dialysis, representing a relatively homogeneous population, and key elements that could influence the analysis—such as dosing regimens and evaluation parameters—were generally similar across studies.*

*Taken together, we consider the impact of inter-trial heterogeneity on the results and conclusions of this analysis to be limited.*

*Based on your suggestion, we have added a description of the main differences among the three trials and a discussion of the potential impact of heterogeneity on the interpretation of the results in the Discussion section, as indicated below.*

***Lines 390-394:***

*The three trials differed in several aspects, which may introduce some degree of heterogeneity; however, the objective of this study was not to compare treatment effects across trials but to exploratorily identify baseline characteristics associated with achieving higher doses.*

2. The analysis was conducted with the “high-dose” versus “low-dose” tenapanor determined based on final dose at Week 8. However, the dose titration was determined based on serum phosphorus levels and tolerability. This may create circular inference.

***Response:*** *Thank you for this important comment. As you pointed out, the classification of high-dose and low-dose groups in this study was a post hoc stratification based on the final dose, and since dose titration was performed based on serum phosphorus levels and tolerability, the possibility of circular inference cannot be fully excluded.*

*However, the aim of this study was not to evaluate causal relationships, but to exploratorily examine which baseline characteristics were associated with patients who ultimately reached higher doses.*

*To address this concern, baseline serum phosphorus levels were included in the multivariable analysis model for adjustment. As a result, serum phosphorus was not identified as an independent predictor in the multivariable analysis.*

*Additionally, the clinical trials included in this study employed a protocol in which serum phosphorus control was achieved through stepwise dose escalation of tenapanor, which differs from real-world clinical practice where multiple phosphate-lowering agents can be flexibly combined or switched. This can be considered a strength of the present study, as it allows for evaluation of tenapanor dose escalation itself.*

*Furthermore, escalation to higher doses depends not only on phosphorus-lowering efficacy but also on tolerability; some patients who require higher doses may not tolerate such escalation in practice.*

*Therefore, this study is characterized by identifying patient characteristics associated with both the need for higher-dose treatment and the ability to reach such doses.*

*In light of these considerations, while the possibility of circular inference represents one of the study limitations, we believe that the comprehensive evaluation of characteristics associated with achieving higher-dose treatment—including tolerability—is of important value. This point has been added to the Discussion section.*

***Lines 406-409:***

*Finally, because dose titration was based on serum phosphorus levels and tolerability, and group classification was based on the final dose, the possibility of circular inference cannot be completely excluded, although baseline serum phosphorus was included in the multivariable analysis.*

3. In statistical analysis, it’s unclear what the model specification is exactly! Model specification should be clearly laid out in the section.

***Response:*** *Thank you for your comment. To identify baseline characteristics associated with the need for higher doses of tenapanor, we defined the outcome variable as achievement of the high-dose group (20 or 30 mg per dose) at Week 8 based on the final dose, and performed both univariate and multivariable logistic regression analyses. The variables included in both univariate and multivariable analyses were prespecified based on clinical relevance. To clarify the explanatory variables used in the analysis, we revised the Statistical Analysis section and added the following description.*

***Lines 210-225:***

*The variables included in the univariate and multivariable analyses were prespecified based on clinical relevance. In the univariate analysis, the following baseline variables were evaluated: sex, age (per 10-year increase), BMI (per 1 kg/m² increase), presence of diabetic nephropathy, dialysis duration (per 1-year increase), dialysis modality (HD, hemodiafiltration [HDF], PD), use of PBs (calcium carbonate, sevelamer hydrochloride, lanthanum carbonate, bixalomer, ferric citrate hydrate, and sucroferric oxyhydroxide), number of PB types (per additional type), serum phosphorus (per 1 mg/dL increase), corrected calcium (per 1 mg/dL increase), intact parathyroid hormone (per 100 pg/mL increase), TRACP-5b (per 100 mU/dL increase), use of concomitant medications (laxatives, acid-suppressing agents, and vitamin D receptor activators), BSFS score and weekly stool frequency (per one increase). In the multivariable analysis, the following prespecified variables were included: sex, age, BMI, presence of diabetic nephropathy, dialysis duration, use of sevelamer hydrochloride, number of PB types, serum phosphorus, intact parathyroid hormone, TRACP-5b, use of concomitant medications (laxatives, acid-suppressing agents, and vitamin D receptor activators), BSFS score and weekly stool frequency.*

4. With current sample size in high-dose group, there is a concern for potential overfitting as it seems that there are 15 predictors in the multivariable model.

***Response:*** *Thank you for raising this concern. Although sample size was taken into consideration, as you pointed out, the relatively large number of explanatory variables compared to the number of patients in the high-dose group means that the possibility of overfitting cannot be completely excluded.*

*We have acknowledged this point as a limitation and added it to the Discussion section.*

***Lines 409-411:***

*Furthermore, the relatively large number of explanatory variables compared with the number of patients in the high-dose group raises the possibility of overfitting.*

5. Dose itself is a time-varying treatment. Please comment on its implications or effect on the results and conclusions.

***Response:*** *Thank you for your comment. As you pointed out, the dose of tenapanor is adjusted during the study period and therefore represents a time-varying exposure.*

*However, the objective of this study was not to analyze temporal dose changes, but to exploratorily identify baseline characteristics of patients who reached high doses at Week 8 as a result of protocol-based dose titration. Therefore, this is unlikely to have materially affected the main findings.*

**Response to Reviewer 2:**

This manuscript presents a pooled analysis of three clinical trials conducted in Japan to identify baseline patient characteristics associated with higher dose requirements of tenapanor in dialysis patients with hyperphosphatemia. The study addresses a clinically relevant and practical question, as dose titration of tenapanor is common in real-world practice yet predictors of higher dose requirements have not been well characterized.

Overall, the manuscript is technically sound, the data support the authors’ conclusions, and the statistical analyses are appropriate and adequately described. The study is presented in a clear and intelligible manner, and the findings provide novel insights that may help inform individualized dosing strategies in clinical practice.

Strengths

Clinical relevance: Identifying factors associated with higher tenapanor dose requirements is highly relevant for nephrologists managing hyperphosphatemia in dialysis patients.

Appropriate methodology: The pooled analysis is reasonable, and the use of both univariate and multivariate analyses to identify independent predictors is appropriate.

Clear results: The separation into low- and high-dose groups is well defined, and baseline comparisons are clearly presented.

Novel findings: The identification of serum TRACP-5b as a positive predictor and BSFS score as a negative predictor of higher dose requirement is novel and biologically plausible, linking bone turnover and bowel habits with tenapanor dose needs.

Balanced discussion: The authors appropriately acknowledge study limitations and the need for confirmation in larger, prospective real-world studies.

***Response:*** *Thank you very much for your positive evaluation. We are honored by your recognition of the clinical relevance of this study, its methodological validity, the clarity of the results, and its novelty in identifying characteristics associated with patients who reached higher doses of tenapanor. Below, we provide point-by-point responses to your comments and suggestions.*

Minor comments / suggestions
The manuscript would benefit from a brief discussion clarifying the potential mechanistic link between elevated TRACP-5b levels and higher tenapanor dose requirements, particularly in relation to bone–mineral metabolism.

***Response:*** *Thank you for this valuable comment. We had already included a discussion of the association between elevated serum TRACP-5b levels and higher tenapanor dose requirements from the perspective of CKD-MBD in the original manuscript (Lines 322–340). In response to your comment, we have further expanded and refined this discussion, as indicated by the underlined text below.*

***Lines 337-360:***

*TRACP-5b is a reliable marker of bone resorption in dialysis patients [23]. Elevated levels reflect increased osteoclast number and activity, often seen in high-turnover bone disease, particularly secondary hyperparathyroidism. Notably, in the present study, parathyroid hormone (a bone formation marker) showed no univariate association with tenapanor dose, whereas TRACP-5b (a bone resorption marker) did.* *In addition, baseline levels of bone-specific alkaline phosphatase, reflecting osteoblast activity, and procollagen type 1 N-terminal propeptide, reflecting collagen production, were largely comparable between the low-dose and high-dose groups. Taken together, these findings support the notion that bone resorption, rather than bone formation, is more closely associated with tenapanor dose requirements. Mechanistically, approximately 85% of total body phosphorus is stored in bone as hydroxyapatite, and osteoclast-mediated resorption releases P into the circulation. Calcimimetics lower serum phosphorus levels by reducing this bone-derived P flux via suppression of parathyroid hormone [24]. By contrast, tenapanor acts by inhibiting intestinal P absorption and does not directly affect bone resorption. Therefore, patients with greater P release from bone, reflected by higher TRACP-5b levels, may require higher tenapanor doses to achieve target serum phosphorus levels. This interpretation is supported by the observation that serum phosphorus levels remained higher in the high-dose group than in the low-dose group throughout the study period. Overall, these results suggest that bone-derived P flux may influence tenapanor dose requirements more than bone formation markers. Clinically, TRACP-5b measurement could help identify dialysis patients needing more intensive serum phosphorus-lowering strategies, including higher tenapanor doses or combination therapy with calcimimetics. Prospective studies integrating bone turnover markers with phosphorus kinetics are warranted.*

Conclusion and recommendation
In conclusion, this study is well conducted, methodologically appropriate, and presents clinically meaningful findings. The data adequately support the conclusions, and the manuscript meets the standards for publication. I recommend acceptance, with only minor editorial revisions if deemed necessary by the journal.

***Response:*** *Thank you very much for your highly positive assessment. We are truly honored that you consider this study worthy of publication.*

**Response to Reviewer 3:**

The paper addresses a practical clinical question — which dialysis patients need higher doses of tenapanor — using data pooled from three phase 3 trials. The two main findings (elevated TRACP-5b and lower BSFS scores predict higher dose requirements) are biologically plausible and modestly supported by the data, but some methodological concerns limit confidence in the conclusions as stated.

***Response:*** *Thank you very much for your constructive evaluation and important comments. While we appreciate your recognition of the clinical significance of this study, your insightful comments regarding methodological concerns were also very valuable. Below, we provide point-by-point responses.*

1. Study design — post-hoc retrospective stratification:
This is the most fundamental issue. Patients were assigned to "low-dose" or "high-dose" groups after 8 weeks of treatment based on where they ended up, not a prospectively defined protocol. This creates inherent confounding: dose escalation was driven by serum phosphorus levels per protocol, so patients with higher baseline phosphorus would mechanically receive higher doses. The high-dose group did indeed have higher baseline phosphorus (7.62 vs 7.24 mg/dL). The authors test phosphorus in the multivariate model but it drops to non-significance (p=0.098), which partly addresses this — but the circularity concern is not fully eliminated and should be discussed more explicitly.

***Response:*** *Thank you for your comment. As you noted, the classification of high- and low-dose groups in this study was based on post hoc stratification according to the final dose, and because dose adjustment was performed based on serum phosphorus levels and tolerability, the issue of circularity cannot be entirely excluded.*

*However, the purpose of this study was not to assess causal relationships, but to exploratorily identify baseline characteristics associated with patients who ultimately reached higher doses.*

*As you correctly pointed out, we accounted for this concern by including baseline serum phosphorus levels in the multivariable analysis model. As a result, serum phosphorus was not identified as an independent predictor.*

*Additionally, the clinical trials included in this study employed a protocol in which serum phosphorus control was achieved through stepwise dose escalation of tenapanor, which differs from real-world clinical practice where multiple phosphate-lowering agents can be flexibly combined or switched. This can be considered a strength of the present study, as it allows for evaluation of tenapanor dose escalation itself.*

*Furthermore, escalation to higher doses depends not only on phosphorus-lowering efficacy but also on tolerability; some patients who require higher doses may not tolerate such escalation in practice.*

*Therefore, this study is characterized by identifying patient characteristics associated with both the need for higher-dose treatment and the ability to reach such doses.*

*In light of these considerations, while the possibility of circular inference represents one of the study limitations, we believe that the comprehensive evaluation of characteristics associated with achieving higher-dose treatment—including tolerability—is of important value. This point has been added to the Discussion section.*

***Lines 406-409:***

*Finally, because dose titration was based on serum phosphorus levels and tolerability, and group classification was based on the final dose, the possibility of circular inference cannot be completely excluded, although baseline serum phosphorus was included in the multivariable analysis.*

2. The three trials differ substantially:
Trial A: HD patients, PB washout before enrollment, placebo-controlled
Trial B: HD patients, ongoing PB combination therapy, blinded phosphorus alerts driving dose changes
Trial C: PD patients (a different clinical population), open-label, 16 weeks

These differences in modality (HD vs PD), concurrent therapy (monotherapy vs combination), blinding, and dose-titration triggers mean the pooled sample is not homogeneous. The authors acknowledge this but dismiss separate analyses due to sample size. This is honest, but reviewers will press on whether pooling here is defensible.

***Response:*** *Thank you for this important comment. We acknowledge that the three phase 3 clinical trials included in this study differ in certain aspects, such as dialysis modality (HD/PD) and the use or washout of phosphate binders, resulting in some degree of heterogeneity across the trials.*

*However, the objective of this study was not to compare treatment effects across trials, but rather to exploratorily identify baseline characteristics common to patients who ultimately reached high doses within clinical trials where dose escalation was conducted stepwise according to protocol.*

*In addition, all trials were conducted in Japanese patients undergoing dialysis, representing a relatively homogeneous population, and key elements that could influence the analysis—such as dosing regimens and evaluation parameters—were generally similar across studies.*

*Taken together, we consider the impact of inter-trial heterogeneity on the results and conclusions of this analysis to be limited.*

*Based on your suggestion, we have added a description of the main differences among the three trials and a discussion of the potential impact of heterogeneity on the interpretation of the results in the Discussion section, as indicated below.*

***Lines 390-394:***

*The three trials differed in several aspects, which may introduce some degree of heterogeneity; however, the objective of this study was not to compare treatment effects across trials but to exploratorily identify baseline characteristics associated with achieving higher doses.*

3. Conflicts of interest:
Two authors (Tokunaga and Asada) are employees of Kyowa Kirin Co., Ltd., the funder. This is disclosed appropriately, but the fact that the sponsoring company's employees performed data curation and investigation is a potential source of bias that a peer reviewer is likely to flag.

***Response:*** *Thank you for your comment. The authors’ affiliations and roles have been disclosed in the Competing Interests and Author Contributions sections.*

*In the revised manuscript, we have also updated the Funding Statement and the Role of Funders in accordance with PLOS ONE requirements, clearly stating that the funder provided support in the form of salaries for ST and AS and was involved in the study design and conduct of the study, as well as in the decision to submit the manuscript. Statistical analyses were performed by an external specialized organization independent of the funder.*

*On the other hand, as the authors are responsible for the analysis plan, data management, and interpretation, their roles are appropriately described as “data curation” and “investigation.”*

***Lines 232-233:***

*All statistical analyses were performed by an external specialized organization, independent of the funder.*
