# Supplementary material for: Patient characteristics associated with the need for high doses of tenapanor hydrochloride in dialysis patients with hyperphosphatemia: A pooled analysis of three clinical trials
Source: PLoS One. 2026 Aug 28;21(8):e0356873. doi: 10.1371/journal.pone.0356873 (PMC13524331; doi:10.1371/journal.pone.0356873)
Supplement: Supplementary file 2 [file pone.0356873.s003.doc]

Dr Emily Chenette

Editor-in-Chief

*PLOS One*

7 Aug 2026

Dear Dr Chenette,

PONE-D-26-10484R1

Please find enclosed our manuscript “Patient characteristics associated with the need for high doses of tenapanor hydrochloride in dialysis patients with hyperphosphatemia: A pooled analysis of three clinical trials,” which we are pleased to resubmit for consideration as an Article in *PLOS One*.

We sincerely appreciate the insightful comments from the reviewers and the opportunity to revise our manuscript. In response to the amendment request, we have carefully addressed all comments and concerns raised during the review process. Detailed point-by-point responses are provided below.

We hope you find our manuscript suitable for publication in *PLOS One* and look forward to hearing from you at your earliest convenience.

Thank you again for your time and consideration. We would be grateful if you would consider our revised manuscript for further evaluation.

Yours sincerely,

Nobuo Nagano

Kidney Disease and Dialysis Center, Hidaka Hospital, Hidaka-kai, 886 Nakao-machi, Takasaki, Gunma, 370-0001, Japan

TEL: +81-27-362-6201

FAX: +81-27-362-8996

E-mail: n_nagano@hidaka-kai.com

**Response to Reviewer 3:**

Minor issues: the discussion text still requires cleaning up — "Mechanistically, Approximately approximately 85% of total body phosphorus..." (line 350-351)

***Response:*** *Thank you for pointing this out. We carefully rechecked the relevant sentence and confirmed that the duplicated word ("approximately") had already been corrected in the revised manuscript submitted for review. Therefore, no additional changes were required.*

Table 3 footnote shows the low-dose group as n=133 while the efficacy tables use n=127 — worth double-checking this is intentional (safety set vs. efficacy/mITT set) and is clearly labeled as such, since it could read as an inconsistency to readers.

***Response:*** *Thank you for this important observation. The difference in sample size is intentional and reflects the use of different analysis populations. Specifically, Table 3 was based on the safety analysis set, whereas the efficacy analyses were conducted using the modified intention-to-treat (mITT) population. To avoid any confusion, we have revised the title of Table 3 to clarify that the analyses were performed in the safety analysis set.*

*Lines 327-329:*

*Table 3. Incidence of gastrointestinal-related adverse drug reactions occurring at a frequency of 5% or higher in the low-dose and high-dose groups in the safety analysis set.^a^*

Pooling heterogeneous trial designs (HD washout/placebo-controlled vs. HD combination-therapy/blinded-alert-driven vs. PD/open-label/16-week) is addressed narratively but not statistically (no trial-stratified analysis or interaction term reported). Consider a sensitivity check showing the TRACP-5b/BSFS associations hold (even descriptively) within each trial (e.g., a forest plot by trial). If possible.

***Response:*** *Thank you for this valuable suggestion. We carefully considered conducting trial-stratified analyses. However, as noted in the Discussion, the sample size of the present study was relatively small even in the pooled analysis. Conducting separate analyses for each trial would further reduce the sample size and potentially result in unstable estimates. Consequently, any observed differences could reflect random variation rather than true associations, making the findings difficult to interpret reliably.*

*Therefore, we decided not to perform additional trial-stratified analyses in the current study. We appreciate the reviewer's understanding of this limitation.*
