# Supplementary material for: Patient characteristics associated with the need for high doses of tenapanor hydrochloride in dialysis patients with hyperphosphatemia: A pooled analysis of three clinical trials
Source: PLoS One. 2026 Aug 28;21(8):e0356873. doi: 10.1371/journal.pone.0356873 (PMC13524331; doi:10.1371/journal.pone.0356873)
Supplement: Peer review history

# Peer review: Decision Letter 0

**DOI:** 10.1371/journal.pone.0356873.r001

**Authors:** Ken Iseri

16 Apr 2026

Dear Dr. Nagano,

Thank you for submitting your manuscript to PLOS ONE. After careful consideration, we feel that it has merit but does not fully meet PLOS ONE’s publication criteria as it currently stands. Therefore, we invite you to submit a revised version of the manuscript that addresses the points raised during the review process.

Please submit your revised manuscript by May 31 2026 11:59PM. If you will need more time than this to complete your revisions, please reply to this message or contact the journal office at plosone@plos.org. When you're ready to submit your revision, log on to https://www.editorialmanager.com/pone/ and select the 'Submissions Needing Revision' folder to locate your manuscript file.

- A letter that responds to each point raised by the academic editor and reviewer(s). You should upload this letter as a separate file labeled 'Response to Reviewers'.
- A marked-up copy of your manuscript that highlights changes made to the original version. You should upload this as a separate file labeled 'Revised Manuscript with Track Changes'.
- An unmarked version of your revised paper without tracked changes. You should upload this as a separate file labeled 'Manuscript'.

If you would like to make changes to your financial disclosure, please include your updated statement in your cover letter. Guidelines for resubmitting your figure files are available below the reviewer comments at the end of this letter.

If applicable, we recommend that you deposit your laboratory protocols in protocols.io to enhance the reproducibility of your results. Protocols.io assigns your protocol its own identifier (DOI) so that it can be cited independently in the future. For instructions see: https://journals.plos.org/plosone/s/submission-guidelines#loc-laboratory-protocols. Additionally, PLOS ONE offers an option for publishing peer-reviewed Lab Protocol articles, which describe protocols hosted on protocols.io. Read more information on sharing protocols at https://plos.org/protocols?utm_medium=editorial-email&utm_source=authorletters&utm_campaign=protocols.

As the corresponding author, your ORCID iD is verified in the submission system and will appear in the published article. PLOS supports the use of ORCID, and we encourage all coauthors to register for an ORCID iD and use it as well. Please encourage your coauthors to verify their ORCID iD within the submission system before final acceptance, as unverified ORCID iDs will not appear in the published article. *Only*  the individual author can complete the verification step; PLOS staff *cannot*  verify ORCID iDs on behalf of authors.

We look forward to receiving your revised manuscript.

Kind regards,

Ken Iseri

Academic Editor

PLOS One

Journal Requirements:

When submitting your revision, we need you to address these additional requirements.

1. Please ensure that your manuscript meets PLOS ONE's style requirements, including those for file naming. The PLOS ONE style templates can be found at

https://journals.plos.org/plosone/s/file?id=wjVg/PLOSOne_formatting_sample_main_body.pdf and

https://journals.plos.org/plosone/s/file?id=ba62/PLOSOne_formatting_sample_title_authors_affiliations.pdf

2. We note that the grant information you provided in the ‘Funding Information’ and ‘Financial Disclosure’ sections do not match.

When you resubmit, please ensure that you provide the correct grant numbers for the awards you received for your study in the ‘Funding Information’ section.

3. Thank you for stating the following in the Competing Interests section:

“Nobuo Nagano received consultancies from Kyowa Kirin Co., Ltd.; honoraria from Kyowa Kirin Co., Ltd.; Kissei Pharmaceutical Co., Ltd.; Toa Shinyaku Co., Ltd.; Nobelpharma Co., Ltd.; Torii Pharmaceutical Co.,Ltd.; and Ono Pharmaceutical Co., Ltd.

Shin Tokunaga and Shinji Asada are employees of Kyowa Kirin Co., Ltd.

Masafumi Fukagawa received grants from Kyowa Kirin Co., Ltd. to his institution; consulting fees and honoraria from Sanwa Kagaku Kenkyusho Co., Ltd.; Ono Pharmaceutical Co., Ltd.; Kyowa Kirin Co., Ltd.; Bayer Yakuhin, Ltd.; Kissei Pharmaceutical Co., Ltd.; and Torii Pharmaceutical Co., Ltd.

Tadao Akizawa received consulting fees from Kyowa Kirin Co., Ltd.; Kissei Pharmaceutical Co., Ltd.; Torii Pharmaceutical Co., Ltd.; and Sanwa Kagaku Kenkyusho Co., Ltd.; honoraria from Kyowa Kirin Co., Ltd.; Kissei Pharmaceutical Co., Ltd.; Ono Pharmaceutical Co., Ltd.; Torii Pharmaceutical Co., Ltd.; and Sanwa Kagaku Kenkyusho Co., Ltd.; support for travel fee from Kyowa Kirin Co., Ltd.”

We note that one or more of the authors are employed by a commercial company: Kyowa Kirin Co., Ltd.; Kissei Pharmaceutical Co., Ltd.; Toa Shinyaku Co., Ltd.; Nobelpharma Co., Ltd.; Torii Pharmaceutical Co.,Ltd.; and Ono Pharmaceutical Co., Ltd.  And Sanwa Kagaku Kenkyusho Co., Ltd.;

1. Please provide an amended Funding Statement declaring this commercial affiliation, as well as a statement regarding the Role of Funders in your study. If the funding organization did not play a role in the study design, data collection and analysis, decision to publish, or preparation of the manuscript and only provided financial support in the form of authors' salaries and/or research materials, please review your statements relating to the author contributions, and ensure you have specifically and accurately indicated the role(s) that these authors had in your study. You can update author roles in the Author Contributions section of the online submission form.

Please also include the following statement within your amended Funding Statement.

“The funder provided support in the form of salaries for authors [insert relevant initials], but did not have any additional role in the study design, data collection and analysis, decision to publish, or preparation of the manuscript. The specific roles of these authors are articulated in the ‘author contributions’ section.”

If your commercial affiliation did play a role in your study, please state and explain this role within your updated Funding Statement.

2. Please also provide an updated Competing Interests Statement declaring this commercial affiliation along with any other relevant declarations relating to employment, consultancy, patents, products in development, or marketed products, etc.

Within your Competing Interests Statement, please confirm that this commercial affiliation does not alter your adherence to all PLOS ONE policies on sharing data and materials by including the following statement: "This does not alter our adherence to  PLOS ONE policies on sharing data and materials.” (as detailed online in our guide for authors http://journals.plos.org/plosone/s/competing-interests) . If this adherence statement is not accurate and  there are restrictions on sharing of data and/or materials, please state these. Please note that we cannot proceed with consideration of your article until this information has been declared.

Please include both an updated Funding Statement and Competing Interests Statement in your cover letter. We will change the online submission form on your behalf.

4. Your ethics statement should only appear in the Methods section of your manuscript. If your ethics statement is written in any section besides the Methods, please move it to the Methods section and delete it from any other section. Please ensure that your ethics statement is included in your manuscript, as the ethics statement entered into the online submission form will not be published alongside your manuscript.

5. If the reviewer comments include a recommendation to cite specific previously published works, please review and evaluate these publications to determine whether they are relevant and should be cited. There is no requirement to cite these works unless the editor has indicated otherwise.

Additional Editor Comments:

Please address reviewer's comments.

[Note: HTML markup is below. Please do not edit.]

Reviewers' comments:

Reviewer's Responses to Questions

**Comments to the Author**

1. Is the manuscript technically sound, and do the data support the conclusions?

Reviewer #1: Yes

Reviewer #2: Yes

Reviewer #3: Yes

**********

2. Has the statistical analysis been performed appropriately and rigorously?

Reviewer #1: No

Reviewer #2: Yes

Reviewer #3: I Don't Know

**********

3. Have the authors made all data underlying the findings in their manuscript fully available?

The PLOS Data policy

Reviewer #1: Yes

Reviewer #2: Yes

Reviewer #3: Yes

**********

4. Is the manuscript presented in an intelligible fashion and written in standard English?

Reviewer #1: Yes

Reviewer #2: Yes

Reviewer #3: Yes

**********

Reviewer #1: The team pooled data from three trials to characterize the sample associated with the need for higher tenapanor doses. The results showed that the serum TRACP-5b and BSFS score as important predictors.

1. This study pooled data from three clinical trials with heterogeneous designs. However, there is no evaluation or discussion on its effect on the results or conclusions.

2. The analysis was conducted with the “high-dose” versus “low-dose” tenapanor determined based on final dose at Week 8. However, the dose titration was determined based on serum phosphorus levels and tolerability. This may create circular inference.

3. In statistical analysis, it’s unclear what the model specification is exactly! Model specification should be clearly laid out in the section.

4. With current sample size in high-dose group, there is a concern for potential overfitting as it seems that there are 15 predictors in the multivariable model.

5. Dose itself is a time-varying treatment. Please comment on its implications or effect on the results and conclusions.

Reviewer #2: General assessment

This manuscript presents a pooled analysis of three clinical trials conducted in Japan to identify baseline patient characteristics associated with higher dose requirements of tenapanor in dialysis patients with hyperphosphatemia. The study addresses a clinically relevant and practical question, as dose titration of tenapanor is common in real-world practice yet predictors of higher dose requirements have not been well characterized.

Overall, the manuscript is technically sound, the data support the authors’ conclusions, and the statistical analyses are appropriate and adequately described. The study is presented in a clear and intelligible manner, and the findings provide novel insights that may help inform individualized dosing strategies in clinical practice.

Strengths

Clinical relevance: Identifying factors associated with higher tenapanor dose requirements is highly relevant for nephrologists managing hyperphosphatemia in dialysis patients.

Appropriate methodology: The pooled analysis is reasonable, and the use of both univariate and multivariate analyses to identify independent predictors is appropriate.

Clear results: The separation into low- and high-dose groups is well defined, and baseline comparisons are clearly presented.

Novel findings: The identification of serum TRACP-5b as a positive predictor and BSFS score as a negative predictor of higher dose requirement is novel and biologically plausible, linking bone turnover and bowel habits with tenapanor dose needs.

Balanced discussion: The authors appropriately acknowledge study limitations and the need for confirmation in larger, prospective real-world studies.

Minor comments / suggestions

The manuscript would benefit from a brief discussion clarifying the potential mechanistic link between elevated TRACP-5b levels and higher tenapanor dose requirements, particularly in relation to bone–mineral metabolism.

Conclusion and recommendation

In conclusion, this study is well conducted, methodologically appropriate, and presents clinically meaningful findings. The data adequately support the conclusions, and the manuscript meets the standards for publication. I recommend acceptance, with only minor editorial revisions if deemed necessary by the journal.

Reviewer #3: The paper addresses a practical clinical question — which dialysis patients need higher doses of tenapanor — using data pooled from three phase 3 trials. The two main findings (elevated TRACP-5b and lower BSFS scores predict higher dose requirements) are biologically plausible and modestly supported by the data, but some methodological concerns limit confidence in the conclusions as stated.

1. Study design — post-hoc retrospective stratification:

This is the most fundamental issue. Patients were assigned to "low-dose" or "high-dose" groups after 8 weeks of treatment based on where they ended up, not a prospectively defined protocol. This creates inherent confounding: dose escalation was driven by serum phosphorus levels per protocol, so patients with higher baseline phosphorus would mechanically receive higher doses. The high-dose group did indeed have higher baseline phosphorus (7.62 vs 7.24 mg/dL). The authors test phosphorus in the multivariate model but it drops to non-significance (p=0.098), which partly addresses this — but the circularity concern is not fully eliminated and should be discussed more explicitly.

2. The three trials differ substantially:

Trial A: HD patients, PB washout before enrollment, placebo-controlled

Trial B: HD patients, ongoing PB combination therapy, blinded phosphorus alerts driving dose changes

Trial C: PD patients (a different clinical population), open-label, 16 weeks

These differences in modality (HD vs PD), concurrent therapy (monotherapy vs combination), blinding, and dose-titration triggers mean the pooled sample is not homogeneous. The authors acknowledge this but dismiss separate analyses due to sample size. This is honest, but reviewers will press on whether pooling here is defensible.

3. Conflicts of interest:

Two authors (Tokunaga and Asada) are employees of Kyowa Kirin Co., Ltd., the funder. This is disclosed appropriately, but the fact that the sponsoring company's employees performed data curation and investigation is a potential source of bias that a peer reviewer is likely to flag.

**********

what does this mean?). If published, this will include your full peer review and any attached files.

If you choose “no”, your identity will remain anonymous but your review may still be made public.

**Do you want your identity to be public for this peer review?**  For information about this choice, including consent withdrawal, please see our Privacy Policy

Reviewer #1: No

Reviewer #2: No

Reviewer #3: No

**********

[NOTE: If reviewer comments were submitted as an attachment file, they will be attached to this email and accessible via the submission site. Please log into your account, locate the manuscript record, and check for the action link "View Attachments". If this link does not appear, there are no attachment files.]

To ensure your figures meet our technical requirements, please review our figure guidelines: https://journals.plos.org/plosone/s/figures

You may also use PLOS’s free figure tool, NAAS, to help you prepare publication quality figures: https://journals.plos.org/plosone/s/figures#loc-tools-for-figure-preparation.

NAAS will assess whether your figures meet our technical requirements by comparing each figure against our figure specifications.

# Author comment: Author response to Decision Letter 1

**DOI:** 10.1371/journal.pone.0356873.r002

10 Jun 2026

Response to Editor:

1. Please ensure that your manuscript meets PLOS ONE's style requirements, including those for file naming. The PLOS ONE style templates can be found at https://journals.plos.org/plosone/s/file?id=wjVg/PLOSOne_formatting_sample_main_body.pdf and https://journals.plos.org/plosone/s/file?id=ba62/PLOSOne_formatting_sample_title_authors_affiliations.pdf

Response: Thank you for your comment. In preparation for resubmission, we revised the manuscript text in accordance with the PLOS ONE style template.

2. We note that the grant information you provided in the ‘Funding Information’ and ‘Financial Disclosure’ sections do not match.

When you resubmit, please ensure that you provide the correct grant numbers for the awards you received for your study in the ‘Funding Information’ section.

Response: Thank you for your comment. We have reviewed and revised the statements in the “Funding Information” and “Financial Disclosure” sections.

3. Thank you for stating the following in the Competing Interests section:

“Nobuo Nagano received consultancies from Kyowa Kirin Co., Ltd.; honoraria from Kyowa Kirin Co., Ltd.; Kissei Pharmaceutical Co., Ltd.; Toa Shinyaku Co., Ltd.; Nobelpharma Co., Ltd.; Torii Pharmaceutical Co.,Ltd.; and Ono Pharmaceutical Co., Ltd.

Shin Tokunaga and Shinji Asada are employees of Kyowa Kirin Co., Ltd.

Masafumi Fukagawa received grants from Kyowa Kirin Co., Ltd. to his institution; consulting fees and honoraria from Sanwa Kagaku Kenkyusho Co., Ltd.; Ono Pharmaceutical Co., Ltd.; Kyowa Kirin Co., Ltd.; Bayer Yakuhin, Ltd.; Kissei Pharmaceutical Co., Ltd.; and Torii Pharmaceutical Co., Ltd.

Tadao Akizawa received consulting fees from Kyowa Kirin Co., Ltd.; Kissei Pharmaceutical Co., Ltd.; Torii Pharmaceutical Co., Ltd.; and Sanwa Kagaku Kenkyusho Co., Ltd.; honoraria from Kyowa Kirin Co., Ltd.; Kissei Pharmaceutical Co., Ltd.; Ono Pharmaceutical Co., Ltd.; Torii Pharmaceutical Co., Ltd.; and Sanwa Kagaku Kenkyusho Co., Ltd.; support for travel fee from Kyowa Kirin Co., Ltd.”

We note that one or more of the authors are employed by a commercial company: Kyowa Kirin Co., Ltd.; Kissei Pharmaceutical Co., Ltd.; Toa Shinyaku Co., Ltd.; Nobelpharma Co., Ltd.; Torii Pharmaceutical Co.,Ltd.; and Ono Pharmaceutical Co., Ltd. And Sanwa Kagaku Kenkyusho Co., Ltd.;

1. Please provide an amended Funding Statement declaring this commercial affiliation, as well as a statement regarding the Role of Funders in your study. If the funding organization did not play a role in the study design, data collection and analysis, decision to publish, or preparation of the manuscript and only provided financial support in the form of authors' salaries and/or research materials, please review your statements relating to the author contributions, and ensure you have specifically and accurately indicated the role(s) that these authors had in your study. You can update author roles in the Author Contributions section of the online submission form.

Please also include the following statement within your amended Funding Statement.

“The funder provided support in the form of salaries for authors [insert relevant initials], but did not have any additional role in the study design, data collection and analysis, decision to publish, or preparation of the manuscript. The specific roles of these authors are articulated in the ‘author contributions’ section.”

If your commercial affiliation did play a role in your study, please state and explain this role within your updated Funding Statement.

Response: Thank you for your comment. We have reviewed the requirements regarding commercial affiliations and the role of funders and have revised the submission information accordingly.

In the revised Funding Statement, we have clarified that the funder provided support in the form of salaries for ST and AS and was involved in the study design and conduct of the study, as well as in the decision to submit the manuscript. Statistical analyses were performed by an external specialized organization independent of the funder. The specific roles of these authors are articulated in the ‘author contributions’ section.

2. Please also provide an updated Competing Interests Statement declaring this commercial affiliation along with any other relevant declarations relating to employment, consultancy, patents, products in development, or marketed products, etc.

Within your Competing Interests Statement, please confirm that this commercial affiliation does not alter your adherence to all PLOS ONE policies on sharing data and materials by including the following statement: "This does not alter our adherence to PLOS ONE policies on sharing data and materials.” (as detailed online in our guide for authors http://journals.plos.org/plosone/s/competing-interests) . If this adherence statement is not accurate and there are restrictions on sharing of data and/or materials, please state these. Please note that we cannot proceed with consideration of your article until this information has been declared.

Please include both an updated Funding Statement and Competing Interests Statement in your cover letter. We will change the online submission form on your behalf.

Response: Thank you for your comment. We have reviewed the requirements for the declaration of competing interests and prepared an updated Competing Interests Statement. We have also included the following required wording in the Competing Interests Statement:

“This does not alter our adherence to PLOS ONE policies on sharing data and materials.”

4. Your ethics statement should only appear in the Methods section of your manuscript. If your ethics statement is written in any section besides the Methods, please move it to the Methods section and delete it from any other section. Please ensure that your ethics statement is included in your manuscript, as the ethics statement entered into the online submission form will not be published alongside your manuscript.

Response: Thank you for your comment. We have reviewed the ethics statement. The content that had been placed outside the Methods section was moved into the Methods section of the manuscript and revised accordingly.

Lines 103-107:

Ethical approval of each clinical trial [18-20] was obtained from the ethics committees of all 94 participating institutions, and written informed consent was obtained from each patient prior to participation. This pooled post hoc analysis was approved by the ethics committee of Kyowa Kirin Co., Ltd (approval number: MA2024_006_0).

5. If the reviewer comments include a recommendation to cite specific previously published works, please review and evaluate these publications to determine whether they are relevant and should be cited. There is no requirement to cite these works unless the editor has indicated otherwise.

Response: Thank you for your comment. We have reviewed the reviewer comments and found no explicit recommendations to cite specific previously published works.

Response to Reviewer 1:

1. This study pooled data from three clinical trials with heterogeneous designs. However, there is no evaluation or discussion on its effect on the results or conclusions.

Response: Thank you for this important comment. We acknowledge that the three phase 3 clinical trials included in this study differ in certain aspects, such as dialysis modality (HD/PD) and the use or washout of phosphate binders, resulting in some degree of heterogeneity across the trials. However, the objective of this study was not to compare treatment effects across trials, but rather to exploratorily identify baseline characteristics common to patients who ultimately reached high doses within clinical trials where dose escalation was conducted stepwise according to protocol.

In addition, all trials were conducted in Japanese patients undergoing dialysis, representing a relatively homogeneous population, and key elements that could influence the analysis—such as dosing regimens and evaluation parameters—were generally similar across studies.

Taken together, we consider the impact of inter-trial heterogeneity on the results and conclusions of this analysis to be limited.

Based on your suggestion, we have added a description of the main differences among the three trials and a discussion of the potential impact of heterogeneity on the interpretation of the results in the Discussion section, as indicated below.

Lines 390-394:

The three trials differed in several aspects, which may introduce some degree of heterogeneity; however, the objective of this study was not to compare treatment effects across trials but to exploratorily identify baseline characteristics associated with achieving higher doses.

2. The analysis was conducted with the “high-dose” versus “low-dose” tenapanor determined based on final dose at Week 8. However, the dose titration was determined based on serum phosphorus levels and tolerability. This may create circular inference.

Response: Thank you for this important comment. As you pointed out, the classification of high-dose and low-dose groups in this study was a post hoc stratification based on the final dose, and since dose titration was performed based on serum phosphorus levels and tolerability, the possibility of circular inference cannot be fully excluded.

However, the aim of this study was not to evaluate causal relationships, but to exploratorily examine which baseline characteristics were associated with patients who ultimately reached higher doses.

To address this concern, baseline serum phosphorus levels were included in the multivariable analysis model for adjustment. As a result, serum phosphorus was not identified as an independent predictor in the multivariable analysis.

Additionally, the clinical trials included in this study employed a protocol in which serum phosphorus control was achieved through stepwise dose escalation of tenapanor, which differs from real-world clinical practice where multiple phosphate-lowering agents can be flexibly combined or switched. This can be considered a strength of the present study, as it allows for evaluation of tenapanor dose escalation itself.

Furthermore, escalation to higher doses depends not only on phosphorus-lowering efficacy but also on tolerability; some patients who require higher doses may not tolerate such escalation in practice.

Therefore, this study is characterized by identifying patient characteristics associated with both the need for higher-dose treatment and the ability to reach such doses.

In light of these considerations, while the possibility of circular inference represents one of the study limitations, we believe that the comprehensive evaluation of characteristics associated with achieving higher-dose treatment—including tolerability—is of important value. This point has been added to the Discussion section.

Lines 406-409:

Finally, because dose titration was based on serum phosphorus levels and tolerability, and group classification was based on the final dose, the possibility of circular inference cannot be completely excluded, although baseline serum phosphorus was included in the multivariable analysis.

3. In statistical analysis, it’s unclear what the model specification is exactly! Model specification should be clearly laid out in the section.

Response: Thank you for your comment. To identify baseline characteristics associated with the need for higher doses of tenapanor, we defined the outcome variable as achievement of the high-dose group (20 or 30 mg per dose) at Week 8 based on the final dose, and performed both univariate and multivariable logistic regression analyses. The variables included in both univariate and multivariable analyses were prespecified based on clinical relevance. To clarify the explanatory variables used in the analysis, we revised the Statistical Analysis section and added the following description.

Lines 210-225:

The variables included in the univariate and multivariable analyses were prespecified based on clinical relevance. In the univariate analysis, the following baseline variables were evaluated: sex, age (per 10-year increase), BMI (per 1 kg/m² increase), presence of diabetic nephropathy, dialysis duration (per 1-year increase), dialysis modality (HD, hemodiafiltration [HDF], PD), use of PBs (calcium carbonate, sevelamer hydrochloride, lanthanum carbonate, bixalomer, ferric citrate hydrate, and sucroferric oxyhydroxide), number of PB types (per additional type), serum phosphorus (per 1 mg/dL increase), corrected calcium (per 1 mg/dL increase), intact parathyroid hormone (per 100 pg/mL increase), TRACP-5b (per 100 mU/dL increase), use of concomitant medications (laxatives, acid-suppressing agents, and vitamin D receptor activators), BSFS score and weekly stool frequency (per one increase). In the multivariable analysis, the following prespecified variables were included: sex, age, BMI, presence of diabetic nephropathy, dialysis duration, use of sevelamer hydrochloride, number of PB types, serum phosphorus, intact parathyroid hormone, TRACP-5b, use of concomitant medications (laxatives, acid-suppressing agents, and vitamin D receptor activators), BSFS score and weekly stool frequency.

4. With current sample size in high-dose group, there is a concern for potential overfitting as it seems that there are 15 predictors in the multivariable model.

Response: Thank you for raising this concern. Although sample size was taken into consideration, as you pointed out, the relatively large number of explanatory variables compared to the number of patients in the high-dose group means that the possibility of overfitting cannot be completely excluded.

We have acknowledged this point as a limitation and added it to the Discussion section.

Lines 409-411:

Furthermore, the relatively large number of explanatory variables compared with the number of patients in the high-dose group raises the possibility of overfitting.

5. Dose itself is a time-varying treatment. Please comment on its implications or effect on the results and conclusions.

Response: Thank you for your comment. As you pointed out, the dose of tenapanor is adjusted during the study period and therefore represents a time-varying exposure.

However, the objective of this study was not to analyze temporal dose changes, but to exploratorily identify baseline characteristics of patients who reached high doses at Week 8 as a result of protocol-based dose titration. Therefore, this is unlikely to have materially affected the main findings.

Response to Reviewer 2:

This manuscript presents a pooled analysis of three clinical trials conducted in Japan to identify baseline patient characteristics associated with higher dose requirements of tenapanor in dialysis patients with hyperphosphatemia. The study addresses a clinically relevant and practical question, as dose titration of tenapanor is common in real-world practice yet predictors of higher dose requirements have not been well characterized.

Overall, the manuscript is technically sound, the data support the authors’ conclusions, and the statistical analyses are appropriate and adequately described. The study is presented in a clear and intelligible manner, and the findings provide novel insights that may help inform individualized dosing strategies in clinical practice.

Strengths

Clinical relevance: Identifying factors associated with higher tenapanor dose requirements is highly relevant for nephrologists managing hyperphosphatemia in dialysis patients.

Appropriate methodology: The pooled analysis is reasonable, and the use of both univariate and multivariate analyses to identify independent predictors is appropriate.

Clear results: The separation into low- and high-dose groups is well defined, and baseline comparisons are clearly presented.

Novel findings: The identification of serum TRACP-5b as a positive predictor and BSFS score as a negative predictor of higher dose requirement is novel and biologically plausible, linking bone turnover and bowel habits with tenapanor dose needs.

Balanced discussion: The authors appropriately acknowledge study limitations and the need for confirmation in larger, prospective real-world studies.

Response: Thank you very mu

Attachment Submitted filename: Response to Reviewers_PONE-D-26-10484.doc

# Peer review: Decision Letter 1

**DOI:** 10.1371/journal.pone.0356873.r003

**Authors:** Ken Iseri

10 Jul 2026

Dear Dr. Nagano,

Thank you for submitting your manuscript to PLOS ONE. After careful consideration, we feel that it has merit but does not fully meet PLOS ONE’s publication criteria as it currently stands. Therefore, we invite you to submit a revised version of the manuscript that addresses the points raised during the review process.

==============================

**ACADEMIC EDITOR:**  Please address the reviewers’ comments.

==============================

Please submit your revised manuscript by Aug 24 2026 11:59PM. If you will need more time than this to complete your revisions, please reply to this message or contact the journal office at plosone@plos.org. When you're ready to submit your revision, log on to https://www.editorialmanager.com/pone/ and select the 'Submissions Needing Revision' folder to locate your manuscript file.

- A letter that responds to each point raised by the academic editor and reviewer(s). You should upload this letter as a separate file labeled 'Response to Reviewers'.
- A marked-up copy of your manuscript that highlights changes made to the original version. You should upload this as a separate file labeled 'Revised Manuscript with Track Changes'.
- An unmarked version of your revised paper without tracked changes. You should upload this as a separate file labeled 'Manuscript'.

If you would like to make changes to your financial disclosure, please include your updated statement in your cover letter. Guidelines for resubmitting your figure files are available below the reviewer comments at the end of this letter.

If applicable, we recommend that you deposit your laboratory protocols in protocols.io to enhance the reproducibility of your results. Protocols.io assigns your protocol its own identifier (DOI) so that it can be cited independently in the future. For instructions see: https://journals.plos.org/plosone/s/submission-guidelines#loc-laboratory-protocols. Additionally, PLOS ONE offers an option for publishing peer-reviewed Lab Protocol articles, which describe protocols hosted on protocols.io. Read more information on sharing protocols at https://plos.org/protocols?utm_medium=editorial-email&utm_source=authorletters&utm_campaign=protocols.

As the corresponding author, your ORCID iD is verified in the submission system and will appear in the published article. PLOS supports the use of ORCID, and we encourage all coauthors to register for an ORCID iD and use it as well. Please encourage your coauthors to verify their ORCID iD within the submission system before final acceptance, as unverified ORCID iDs will not appear in the published article. *Only*  the individual author can complete the verification step; PLOS staff *cannot*  verify ORCID iDs on behalf of authors.

We look forward to receiving your revised manuscript.

Kind regards,

Ken Iseri

Academic Editor

PLOS One

Journal Requirements:

1. If the reviewer comments include a recommendation to cite specific previously published works, please review and evaluate these publications to determine whether they are relevant and should be cited. There is no requirement to cite these works unless the editor has indicated otherwise.

2. Please review your reference list to ensure that it is complete and correct. If you have cited papers that have been retracted, please include the rationale for doing so in the manuscript text, or remove these references and replace them with relevant current references. Any changes to the reference list should be mentioned in the rebuttal letter that accompanies your revised manuscript. If you need to cite a retracted article, indicate the article’s retracted status in the References list and also include a citation and full reference for the retraction notice.

[Note: HTML markup is below. Please do not edit.]

Reviewers' comments:

Reviewer's Responses to Questions

**Comments to the Author**

Reviewer #1: All comments have been addressed

Reviewer #3: All comments have been addressed

**********

2. Is the manuscript technically sound, and do the data support the conclusions?

Reviewer #1: (No Response)

Reviewer #3: Yes

**********

3. Has the statistical analysis been performed appropriately and rigorously?

Reviewer #1: (No Response)

Reviewer #3: I Don't Know

**********

4. Have the authors made all data underlying the findings in their manuscript fully available?

The PLOS Data policy

Reviewer #1: (No Response)

Reviewer #3: Yes

**********

5. Is the manuscript presented in an intelligible fashion and written in standard English?

Reviewer #1: (No Response)

Reviewer #3: Yes

**********

Reviewer #1: (No Response)

Reviewer #3: This pooled post-hoc analysis combines data from three Japanese phase 3 clinical trials (n=212) to identify which dialysis patients with hyperphosphatemia are most likely to need higher doses of tenapanor. Patients were retrospectively split into a low-dose group (5–10 mg/dose, n=127) and a high-dose group (20–30 mg/dose, n=85) based on their dose at Week 8, and baseline characteristics were compared using univariate and multivariate logistic regression. The analysis found that higher baseline levels of TRACP-5b and lower Bristol Stool Form Scale scores were independent predictors of needing a higher tenapanor dose. The authors conclude that patients with greater bone turnover or constipation-prone bowel habits are more likely to require dose escalation. They suggest that assessing bone turnover and bowel habits could help guide individualized dosing, while noting that larger, prospective real-world studies are needed to confirm these findings.

Minor issues: the discussion text still requires cleaning up — "Mechanistically, Approximately approximately 85% of total body phosphorus..." (line 350-351)

Table 3 footnote shows the low-dose group as n=133 while the efficacy tables use n=127 — worth double-checking this is intentional (safety set vs. efficacy/mITT set) and is clearly labeled as such, since it could read as an inconsistency to readers.

Pooling heterogeneous trial designs (HD washout/placebo-controlled vs. HD combination-therapy/blinded-alert-driven vs. PD/open-label/16-week) is addressed narratively but not statistically (no trial-stratified analysis or interaction term reported). Consider a sensitivity check showing the TRACP-5b/BSFS associations hold (even descriptively) within each trial (e.g., a forest plot by trial). If possible.

**********

what does this mean?). If published, this will include your full peer review and any attached files.

If you choose “no”, your identity will remain anonymous but your review may still be made public.

**Do you want your identity to be public for this peer review?**  For information about this choice, including consent withdrawal, please see our Privacy Policy

Reviewer #1: No

Reviewer #3: No

**********

[NOTE: If reviewer comments were submitted as an attachment file, they will be attached to this email and accessible via the submission site. Please log into your account, locate the manuscript record, and check for the action link "View Attachments". If this link does not appear, there are no attachment files.]

To ensure your figures meet our technical requirements, please review our figure guidelines: https://journals.plos.org/plosone/s/figures

You may also use PLOS’s free figure tool, NAAS, to help you prepare publication quality figures: https://journals.plos.org/plosone/s/figures#loc-tools-for-figure-preparation.

NAAS will assess whether your figures meet our technical requirements by comparing each figure against our figure specifications.

# Author comment: Author response to Decision Letter 2

**DOI:** 10.1371/journal.pone.0356873.r004

7 Aug 2026

Dr Emily Chenette

Editor-in-Chief

PLOS One

7 Aug 2026

Dear Dr Chenette,

PONE-D-26-10484R1

Please find enclosed our manuscript “Patient characteristics associated with the need for high doses of tenapanor hydrochloride in dialysis patients with hyperphosphatemia: A pooled analysis of three clinical trials,” which we are pleased to resubmit for consideration as an Article in PLOS One.

We sincerely appreciate the insightful comments from the reviewers and the opportunity to revise our manuscript. In response to the amendment request, we have carefully addressed all comments and concerns raised during the review process. Detailed point-by-point responses are provided below.

We hope you find our manuscript suitable for publication in PLOS One and look forward to hearing from you at your earliest convenience.

Thank you again for your time and consideration. We would be grateful if you would consider our revised manuscript for further evaluation.

Yours sincerely,

Nobuo Nagano

Kidney Disease and Dialysis Center, Hidaka Hospital, Hidaka-kai, 886 Nakao-machi, Takasaki, Gunma, 370-0001, Japan

TEL: +81-27-362-6201

FAX: +81-27-362-8996

E-mail: n_nagano@hidaka-kai.com

Attachment Submitted filename: Response to Reviewers.doc

# Editor report: Decision Letter 2

**DOI:** 10.1371/journal.pone.0356873.r005

**Authors:** Ken Iseri

10 Aug 2026

Patient characteristics associated with the need for high doses of tenapanor hydrochloride in dialysis patients with hyperphosphatemia: A pooled analysis of three clinical trials

PONE-D-26-10484R2

Dear Dr. Nagano,

We’re pleased to inform you that your manuscript has been judged scientifically suitable for publication and will be formally accepted for publication once it meets all outstanding technical requirements.

Within one week, you’ll receive an e-mail detailing the required amendments. When these have been addressed, you’ll receive a formal acceptance letter and your manuscript will be scheduled for publication.

An invoice will be generated when your article is formally accepted. Please note, if your institution has a publishing partnership with PLOS and your article meets the relevant criteria, all or part of your publication costs will be covered. Please make sure your user information is up-to-date by logging into Editorial Manager at Editorial Manager® and clicking the ‘Update My Information' link at the top of the page. For questions related to billing, please contact billing support.

If your institution or institutions have a press office, please notify them about your upcoming paper to help maximize its impact. If they’ll be preparing press materials, please inform our press team as soon as possible -- no later than 48 hours after receiving the formal acceptance. Your manuscript will remain under strict press embargo until 2 pm Eastern Time on the date of publication. For more information, please contact onepress@plos.org.

Kind regards,

Ken Iseri

Academic Editor

PLOS One

Additional Editor Comments (optional):

Reviewers' comments:

# Editor report: Acceptance letter

**DOI:** 10.1371/journal.pone.0356873.r006

**Authors:** Ken Iseri

PONE-D-26-10484R2

PLOS One

Dear Dr. Nagano,

I'm pleased to inform you that your manuscript has been deemed suitable for publication in PLOS One. Congratulations! Your manuscript is now being handed over to our production team.

At this stage, our production department will prepare your paper for publication. This includes ensuring the following:

* All references, tables, and figures are properly cited

* All relevant supporting information is included in the manuscript submission,

* There are no issues that prevent the paper from being properly typeset

You will receive further instructions from the production team, including instructions on how to review your proof when it is ready. Please keep in mind that we are working through a large volume of accepted articles, so please give us a few days to review your paper and let you know the next and final steps.

Lastly, if your institution or institutions have a press office, please let them know about your upcoming paper now to help maximize its impact. If they'll be preparing press materials, please inform our press team within the next 48 hours. Your manuscript will remain under strict press embargo until 2 pm Eastern Time on the date of publication. For more information, please contact onepress@plos.org.

You will receive an invoice from PLOS for your publication fee after your manuscript has reached the completed accept phase. If you receive an email requesting payment before acceptance or for any other service, this may be a phishing scheme. Learn how to identify phishing emails and protect your accounts at https://explore.plos.org/phishing.

If we can help with anything else, please email us at customercare@plos.org.

Thank you for submitting your work to PLOS One and supporting open access.

Kind regards,

PLOS ONE Editorial Office Staff

on behalf of

Dr. Ken Iseri

Academic Editor

PLOS One
